# Contrast Adaptation Contributes to Contrast-Invariance of Orientation Tuning of Primate V1 Cells

**DOI:** 10.1371/journal.pone.0004781

**Published:** 2009-03-10

**Authors:** Lionel G. Nowak, Pascal Barone

**Affiliations:** Centre de Recherche Cerveau et Cognition, Université Toulouse 3-CNRS, Toulouse, France; University of Sydney, Australia

## Abstract

**Background:**

Studies in rodents and carnivores have shown that orientation tuning width of single neurons does not change when stimulus contrast is modified. However, in these studies, stimuli were presented for a relatively long duration (e. g., 4 seconds), making it possible that contrast adaptation contributed to contrast-invariance of orientation tuning. Our first purpose was to determine, in marmoset area V1, whether orientation tuning is still contrast-invariant with the stimulation duration is comparable to that of a visual fixation.

**Methodology/Principal Findings:**

We performed extracellular recordings and examined orientation tuning of single-units using static sine-wave gratings that were flashed for 200 msec. Sixteen orientations and three contrast levels, representing low, medium and high values in the range of effective contrasts for each neuron, were randomly intermixed. Contrast adaptation being a slow phenomenon, cells did not have enough time to adapt to each contrast individually. With this stimulation protocol, we found that the tuning width obtained at intermediate contrast was reduced to 89% (median), and that at low contrast to 76%, of that obtained at high contrast. Therefore, when probed with briefly flashed stimuli, orientation tuning is not contrast-invariant in marmoset V1. Our second purpose was to determine whether contrast adaptation contributes to contrast-invariance of orientation tuning. Stationary gratings were presented, as previously, for 200 msec with randomly varying orientations, but the contrast was kept constant within stimulation blocks lasting >20 sec, allowing for adaptation to the single contrast in use. In these conditions, tuning widths obtained at low contrast were still significantly less than at high contrast (median 85%). However, tuning widths obtained with medium and high contrast stimuli no longer differed significantly.

**Conclusions/Significance:**

Orientation tuning does not appear to be contrast-invariant when briefly flashed stimuli vary in both contrast and orientation, but contrast adaptation partially restores contrast-invariance of orientation tuning.

## Introduction

For most neurons in area V1, response amplitude depends on stimulus orientation [e.g., 1], [2]. It is also known that V1 neurons response amplitude depends on stimulus contrast [e.g., 3]–[5]. Following Sclar and Freeman (1982) [6], multiple studies have therefore examined interactions between contrast and orientation selectivity [7]–[13]. All these studies demonstrated that, although response amplitude increases with contrast, the width of orientation-tuning curves remains constant. The contrast-invariance of orientation tuning thus revealed showed the limitations of the purely feedforward model of orientation selectivity, initially proposed by Hubel and Wiesel (1962) [1], which predicts that, through an “iceberg effect”, orientation tuning curves should widen when contrast increases (for a comprehensive account of the “iceberg problem” see [13], [14]). Contrast-invariance of orientation tuning therefore constitutes a strong constraint for understanding mechanisms underlying generation of orientation selectivity and has been the cornerstone in numerous modeling studies attempting to explain generation of orientation tuning [9], [13]–[25].

However, the above studies demonstrating contrast-invariance of orientation tuning have all been performed in carnivores (cat or ferret) or rodents (squirrel). Whether orientation tuning is also contrast-invariant in primate V1 is not firmly established. One study examined orientation selectivity at different contrasts in the primate [26] but did not explicitly report interactions between orientation tuning and contrast. Another study examined contrast-response relationship using drifting gratings that could take 3 different orientations, and concluded that orientation tuning in the macaque is contrast-invariant [27]; however, orientation tuning curves were not formally examined at different contrasts. Finally, one preliminary report described either an effect, or no effect of contrast, depending on the parameters used to quantify orientation tuning [28].

Furthermore, data demonstrating contrast-invariance of orientation tuning were generally obtained with relatively lengthy presentation of drifting sine-wave gratings – for four seconds usually. Contrast adaptation mechanisms could be activated during this time. At the neuronal level, contrast adaptation corresponds to the slow adjustment of firing rates that is observed during the lengthy presentation of a stimulus of constant contrast. For example, contrast adaptation appears as a progressive decline of response amplitude during the presentation of a constant high contrast stimulus [e. g., 29]. Contrast adaptation, as defined here, should not be confounded with “contrast normalization” or “contrast-gain control”, which have different impacts on neuronal properties and which appear to be almost instantaneous [e. g., 30].

It has been shown that, for many V1 cells, the time constant of contrast adaptation is less than 4 seconds [31]–[37]. However, when exploring a visual scene, our eyes constantly move in sequences of fast saccades and short duration fixations. Saccade duration appears to be extremely short, as it is linearly related to the distance between starting and ending points with a rate of 2–3 msec/deg in humans and 1–2 msec/deg in monkeys (e.g., [38], [39]; for review see [40]. When exploring natural scenes or faces, fixations usually last only about 0.2–0.3 second [41]–[44]. Intervals between microsaccades show comparable values [45]. Thus, three to five times per second or so, receptive fields (RFs) of V1 neurons fall upon regions of orientation and contrast that are likely to differ from those previously encountered. Consequently, RFs of neurons in the visual system meet new visual stimuli at a rate of about 3–5 Hz, which is too high to allow for adaptation to the contrast of each stimulus individually.

The first purpose of our study was to determine whether orientation selectivity is contrast-invariant with stimuli varying in *both* contrast and orientation when they are presented for a short duration (0.2 sec), corresponding to that of a fixation. We performed recordings in area V1 of a new world monkey, the common marmoset. The proportion of orientation selective neurons and tuning bandwidths of single-units in marmoset area V1 appear similar to those found in macaque V1 [46]–[48], as does the organization of orientation domains at the columnar level [49], [50]. Our results show that, with this particular stimulation regime, orientation tuning is not contrast-invariant in the primary visual cortex of the marmoset. Tuning curves were on average narrower with lower contrast. This could arise from two differences between the present and previous studies: either the use of a primate instead of carnivore or rodent, or the stimulation regime used. This motivated the second facet of this study. The question we examined is whether contrast adaptation contributes to contrast-invariance of orientation selectivity. Our results show that, even when adapted to a given contrast, orientation tuning curves remained slightly narrower with the lowest contrast compared to the highest contrast in area V1 of the common marmoset. However, contrast adaptation did reduce the difference in orientation tuning observed between different contrasts, indicating that contrast adaptation does contribute to making orientation tuning less contrast-dependent.

## Methods

### Surgical protocol

All procedures were conducted in accordance with the guidelines from the French Ministry of Agriculture (décret 87/848) and from the European Community (directive 86/609) and was approved by the local ethical committee (MP/02/02/01/05, Comité régional d'éthique pour l'expérimentation animal, Midi-Pyrénées). The protocol used for marmoset preparation has been adapted from other published protocols [51]–[53]. Experiments were performed on male and female adult common marmosets (*Callithrix jacchus*, n = 6) weighting 350–450 g. One half hour before anesthesia induction, animals were tranquilized with diazepam (Valium®, Roche) (i. m., 3 mg/kg). At the same time, atropine (0.05 mg/kg) was injected subcutaneously to reduce secretions and to prevent bradycardia. Anesthesia was induced with Alphadalone/Alphaxalone acetate (Saffan®, Essex Pharma, 1.2 ml/kg) injected intramuscularly. Synthetic corticoids Dexamethasone (Merck) or Solumedrol (Pfizer) were given at the same time to prevent brain edema (1 mg/kg). Once anesthetized, animal's body temperature was maintained at 38°C using a heating pad controlled by a rectal thermistor (Homeothermic Blanket System, Harvard Apparatus, USA). EKG recording was performed through metallic pliers. All incision sites were infiltrated with the local anesthetic lidocaïn (Xylocaine®). A venous catheter (OD 0.7 mm, Folioplast, France) was placed in the femoral vein to allow for intravenous infusion of solutions. Anesthesia was maintained during the remainder of the surgery by i. v. Saffan injection (0.17 ml/kg every 10–15 minutes). A tracheotomy was performed and a tracheal tube was inserted to allow artificial ventilation. The marmoset was then set in a stereotaxic frame. A homemade support for eyes and mouth bars has been built (following the design in [51]) to allow fixation of the small marmoset's head. Two holes were drilled over the frontal cortex and Ag wires inserted for epidural EEG recording. A 3–4 mm wide craniotomy was also made to gain access to area V1. A well was constructed using dental cement (Protemp® II) around the V1 craniotomy. A head post was sealed with a screw and dental acrylic (Paladur®, Heraeus, Germany) to the skull and fixed to the stereotaxic apparatus. Once the head firmly held in position, ears, eyes and mouth bars were removed.

Following surgery, the animal was artificially ventilated with N_2_O/O_2_ (50%/50%) using a ventilator (Small Animal Respiration Pump, series 660 & 670, Harvard Apparatus, USA) whose volume and rate were initially set at 12 ml and 30 strokes/min respectively, and adjusted so as to keep end-tidal CO_2_ level, measured with a Capstar-100 Capnometer (CWE, USA), between 4 and 5%. Anesthesia and analgesia were supplemented by a continuous infusion of sufentanil citrate (Sufenta®, Janssen, 4–6 µg/kg/hr) after a loading dose of 1 µg/kg. The infusion vehicle was made of the mixture of 2 ml glucose 30%, 15 ml of amino-acid perfusion solution (Totamin®, Baxter) and included synthetic corticoids (0.4 mg/kg/hr); NaCl was added to a final volume of 50 ml. We waited for 1–2 hours of infusion with this solution to ensure adequate depth of anesthesia. The animal was then paralyzed by adding pancuronium bromide (Pavulon®, Organon, 0.1 mg/kg/hr) to the solution described above.

Mydriasis and cycloplegia were induced with ophthalmic atropine sulfate (1%, Alcon). Gas permeable contact lenses (PMMA, base curve radius 3.4–3.8 mm, base diameter 6 mm, dioptric power 0) were used to protect the eyes. Lenses were cleaned every day and neomycin sulphate (0.25 mg/ml, Sanofi-Aventis) eye drops applied to prevent infection. Optic disks were located using a reversible ophthalmoscope. RF eccentricity was determined relative to the position of the optic disk and, using histological sections, relative to published correlation between recording sites and RF position [54].

Visual stimuli were presented onto a computer monitor placed at 114 cm from the animal's eyes. For improving the focusing of the eyes, we examined responses to high spatial frequency sine-wave gratings and optimized the response by placing corrective lenses in front of the eyes.

The heart rate, rectal temperature and expiratory CO_2_ concentration were monitored throughout the experiment and maintained at 250–350 bpm, 37–38°C and 3–5%, respectively. The EEG and the absence of reaction to noxious stimuli were regularly checked.

### Recording procedure and spike sorting

Action potentials were recorded extracellularly through tungsten in glass microelectrodes [55]. To improve recording stability, the well surrounding the V1 craniotomy was filled with silicone oil (DC 200). Action potentials were acquired with a 1401power interface and Spike2® software (Cambridge Electronic Design, Cambridge, UK) with a digitization rate of 40–50 KHz. The collected signal usually contained spikes from multiple units. Spike sorting was done offline using Spike2's principal component analysis based spike sorting algorithms. Analysis of interspike interval histograms (ISIHs) issued from intracellularly recorded neurons (data set used in [56]) shows that cortical neurons refractory period is >1.5 msec, except in some burst generating neurons (a subpopulation of ‘chattering’ cells with extremely high intraburst frequency). In addition to their shape constancy, extracellularly recorded spikes were therefore considered to be issued from one single neuron if the refractory period, determined from ISIH calculated with a bin width of 0.1 msec, was >1.5 msec – deviation from this criterion was admitted in a few burst-generating neurons with high intraburst frequency.

### Visual stimulation

The location of the RFs was determined with a hand-held projector. Eye preference was then determined and all subsequent visual stimuli were delivered through the dominant eye. Computer controlled stimuli were generated with a VSG2/2F board (CRS, Cambridge, UK) in the initial experiments, and with a VSG Visage system in the last experiments. Scripts for visual stimuli generation and presentation were written in the Matlab environment. Visual stimuli were presented on a Daewoo CMC-2100 ME, 21 inches color monitor (100 Hz non-interlaced refresh, 640×487 resolution) in the initial experiments and on a 22 inches, Mitsubishi Diamond Pro 2070^SB^ color monitor (100 Hz non-interlaced refresh, 800×600 resolution) in the last experiments. Gamma corrections were regularly made to produce accurate stimulus contrast, using VSG's “OptiCAL” photometer and associated automated correction. Contrast corresponds to Michelson's contrast, defined relative to maximal and minimal luminance (*L*_max_ and *L*_min_, respectively) of the gratings as 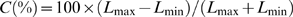.

Cell selectivities and optimal stimuli were evaluated from PSTHs calculated on-line from the multi-unit recording. The preferred orientation of the cell or cells cluster was determined using drifting square-wave gratings presented at eight orientations, each presented in two motion directions (16 stimuli in total, 22.5 deg steps). The grating was presented within a circular patch, 2–6 degrees diameter, centered on the RF. The remaining of the screen was a gray background with a luminance equal to the mean grating luminance. The drift temporal frequency was between 0.5 and 2 cycles/sec. It was qualitatively chosen as the one optimizing the response, as judged by listening to the cell's response on the audio-monitor. To avoid transient responses, the contrast was incremented from 0 to 40% in a 1 sec duration ramp, maintained at 40% for 3 or 4 sec, then decreased back to 0% in a 1 sec duration ramp, then maintained at 0% contrast for 1 sec. The measurement of mean firing rates was restricted to the 3–4 sec plateau period.

Once the preferred orientation was characterized, the preferred spatial frequency was determined using sinusoidal drifting gratings (40% contrast). Drift speed, window size and timing of stimulus presentation were the same as for the orientation tuning protocol. Spatial frequencies varied either between 0.125 cy/deg and 2.83 cy/deg, or between 0.5 and 16 cy/deg in logarithmic steps (increment by 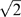).

The response as a function of contrast was then determined, using drifting sinusoidal gratings presented with the orientation and the spatial frequency optimal for the cells under study. Window size, drift rate and stimulus timing were the same as those used for orientation and spatial frequency tuning. Twelve contrasts ranging between 2 and 90% in logarithmic steps (increment by 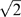) were presented. Contrast-response functions (CRFs) were computed on-line from the multi-unit recording.

From the CRFs, three contrast values were extracted: one causing approximately 80–90% of the maximal response (“high contrast”), one causing 20–25% of the maximal response (“low contrast”), and one causing approximately 50% of the maximal response (“medium contrast”).

**Figure 1:**
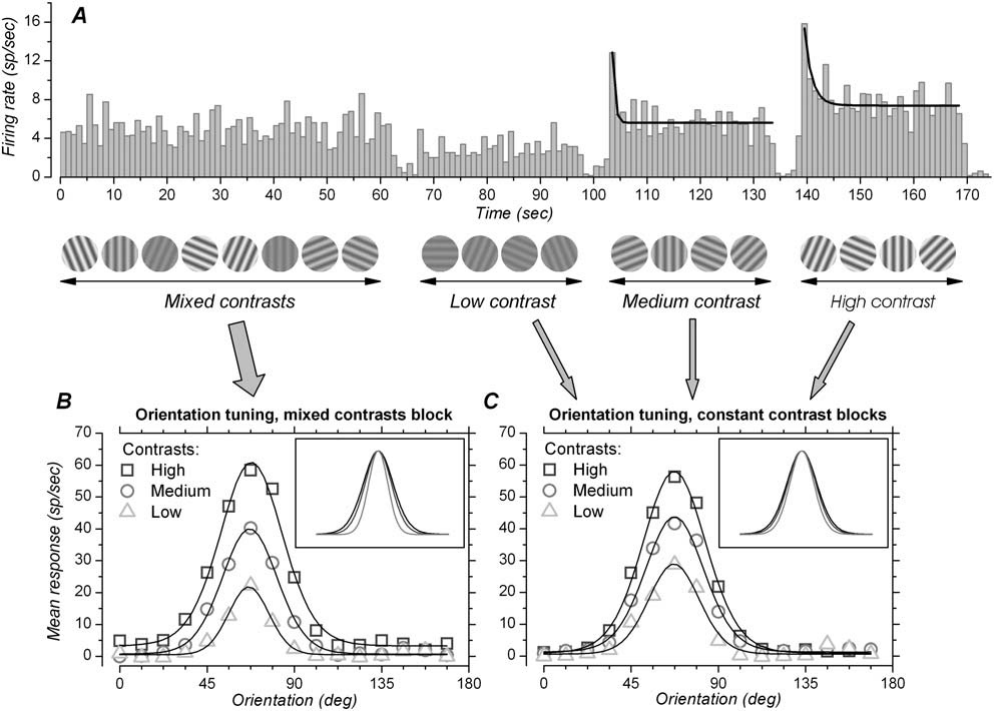
Protocol and orientation tuning with different contrasts, with and without matched adaptation, example. A. PSTH (bin width 1 sec) of the spiking response obtained with the four blocks of stimuli repeated 12 times in a marmoset V1 cell. Some of the grating stimuli, varying in contrast and orientation, are sketched below the PSTH. Sixteen orientations (from 0 to 168.75 deg, 11.25 deg steps) and 3 contrasts (16, 32 and 64% for this neuron) were randomly presented during the “mixed contrasts” block. Each grating presentation was 0.2 sec long, which is too short to allow for contrast adaptation. Since contrast varies at high rate, adaptation can only occur for a contrast level which is the mean of the different contrasts presented: there is a *mismatch* between the stimulus contrast presented at a particular time, and the contrast to which the cell is adapted. For the second, third and fourth stimulation blocks, the 16 orientations were still randomly presented, but only one contrast at a time was used: either low, medium or high. The duration of each block (>30 sec in this example) was long enough to allow for adaptation to each of the contrasts. The stimulus contrast presented at a particular time then *matched* the contrast to which the cell was adapted. Black lines on medium and high contrast responses correspond to the exponential decay fitted to the data. The time constant of adaptation was 0.54 sec with the medium contrast and 1.42 sec with the high contrast. There was no significant adaptation with the low contrast. B. Orientation tuning for data obtained during the mixed contrasts block. Symbols correspond to the mean firing rate for each orientation and contrast, and the lines correspond to the von Mises equation fitted to the orientation-response data. Inset shows fitted lines normalized to the same preferred orientation and to the same height, to facilitate comparison of tuning width. HWHH were 19.0, 16.8 and 12.2 deg for the tuning curves obtained with high, medium and low contrast stimuli, respectively. C. As in B, but for responses obtained after adaptation to either low, medium or high contrast. Spikes outside steady state adaptation, considered to begin at a time corresponding to 3 times the adaptation time constant, were not included in the calculation. HWHH were 19.2, 17.9 and 15.4 deg for the tuning curves obtained with the high, medium and low contrast stimuli, respectively. In this cell, adaptation led to a compression of the range of tuning widths and response amplitudes obtained with the different contrasts. The discrepancy between the spike rates in the PSTH in A and the orientation tuning curves in B and C is due to the fact that interstimulus intervals and responses to non-preferred orientations are included in the average for the long time-scale PSTH.

Our first aim was to determine whether orientation tuning is contrast-invariant with briefly flashed stimuli. Our second aim was to determine the consequences of contrast adaptation on orientation selectivity. The stimulation protocol we used to fulfill these aims is depicted on Fig. 1. Stimuli were stationary sine-wave gratings that were flashed for 200 msec, followed by a blank screen (0% contrast, mean luminance identical to that of grating stimuli) lasting 200–400 msec. Stimuli were presented in a 2 to 6 deg wide circular window. These diameters were deliberately larger than the hand mapped RFs. The RFs size (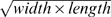) was on average 0.4 deg (range: 0.1–0.8 deg) for opercular recordings (eccentricity <3 deg) and 1.2 deg (range: 0.3–2.7 deg) for calcarine recordings (eccentricity between 6 and 16 deg), in agreement with values reported previously for marmoset V1 [57]. However, it has been shown that low contrast stimuli result in increase of neurons summation area [58]–[60]. We therefore used stimuli that were, on average, 9 times larger than the RF, in order to be sure that the RF would be entirely covered by the stimulus, including when using low contrast. We tried to keep the stimulus size proportional to the RF size, which resulted in stimuli that were larger for calcarine compared to opercular recordings (medians: 5 and 3 deg, respectively). The spatial frequency of the grating was the one determined to be optimal for the cells under study. The phase of the grating varied randomly and could take 4 or 8 different values (increment 2π/4 or 2π/8). The orientation varied randomly from one presentation to the next and could take 16 different values between 0 deg and 168.75 deg (11.25 deg steps).

In the first block of stimulus presentation (Fig. 1A, left, “mixed contrasts”), the contrast of the grating could take, randomly, one of the three values (low, medium and high contrast) determined from the CRF. Thus in this first block, that lasted 40 sec at least, both orientations and contrasts varied randomly. Randomization protocol was “blockwise”, with no repeats of a given stimulus until all 48 stimuli have been presented. Contrast adaptation is a relatively slow phenomenon (>200 msec; [31]–[37]), and it could not occur for each contrast during this first block. However, adaptation probably occurred for a contrast value representing the mean of the three contrasts in use. The long time course of contrast adaptation therefore resulted in a *mismatch* between the contrast presented at one particular moment and the contrast to which the cell was adapted.

In the second, third and fourth stimulus presentation blocks, orientation still varied randomly (blockwise randomization), but the contrast within each block was fixed: in the second block to low contrast only, in the third block to medium contrast only, and in the fourth block to high contrast only (Fig. 1A, “low”, “medium” and “high contrast”). These blocks correspond to the “constant contrast” conditions. Since each block lasted at least 20 sec and since contrast adaptation supposedly has a time constant of seconds, neurons had enough time to adapt to the unique contrast used in each block. The contrast presented at any time and the adapting contrast did *match* in this condition. Stimulus presentation time and interstimulus interval were the same as for the first block.

### Data analysis

All analyses were done off-line after single-unit isolation.

#### Adaptation during the presentation of constant contrast

Presence or lack of firing rate adaptation during the presentation of high, medium or low contrast stimuli was determined using Abeles' method [61], based on confidence intervals calculated on spike counts. For this purpose, we calculated a PSTH for each contrast with a bin width of 5 sec. Time 0 corresponds to the beginning of a block. The mean spike count, *x*, for the first bin was used to calculate the 95% confidence limits, *L_95%_*, using the formula:
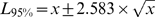



Neurons were considered to show significant adaptation when the mean spike count in the fourth bin (15–20 sec) was less than the lower 95% confidence limit. We also considered the possibility that neurons may be “accelerating”, that is, that the spike count in the fourth bin was larger than the upper 95% limit – but this occurred in only a small number of cases (n = 2/69 cells with low contrast and n = 2/105 cells with medium contrast).

#### Time course of adaptation

In cells that showed significant adaptation, we next evaluated the time constant of adaptation. For this purpose, PSTHs were calculated for each contrast with a bin width of 0.8–1.2 sec (this corresponds to twice the grating presentation period). The data (firing rate vs. time) were then fit with a single exponential (Fig. 1A). Time constants were not further considered when their value was less than their associated standard errors.

The time constant of adaptation was used to delineate a period corresponding to the adapted state for the analysis of orientation selectivity with constant contrast conditions: adaptation was considered to have reached a steady state at a time corresponding to three times the time constant of adaptation. In cells with accelerating responses, the first 5 sec of the response were excluded from orientation tuning calculation. We also excluded the first 5 sec of the blocks in cells that showed a significant adaptation but for which we could not fit the data satisfactorily. The first 5–10 sec of the mixed contrasts block were also excluded from orientation tuning calculation.

#### Orientation tuning

Single-unit spike trains were transformed into spike density functions: each spike was replaced with a raised cosine waveform (half-width: 10 msec). The sampling interval for the spike density was 5 msec. Averages of the spike density function, collapsing all spatial phases, were calculated for each orientation and for each of the 6 stimulus conditions separately. This resulted in 6 sets of orientation tuning curves: for low, medium and high contrast in the mixed contrasts condition, for low, medium and high contrast in the constant contrast condition, restricted to the adapted response only.

Mean spontaneous activity was delineated between stimulus onset (time 0) and 200 msec prior to stimulus onset (adjustments down to 100 msec were required if the neuron showed an appreciable ‘off’ response). Significance of the responses was determined relative to the distribution of spontaneous activity bins amplitude (bin width 5 msec). Responses were considered ‘significant’ if their amplitudes were larger than 1.5 times the highest bin in the spontaneous activity period in two consecutive bins. This approach allowed us to dismiss false positives regardless of the statistics underlying spontaneous activity amplitude distribution. This arbitrary criterion is a very conservative one: if the noise was distributed in a Gaussian manner, then the p value associated with our criterion would be extremely low (p≪0.01).

Mean firing rate for each orientation was calculated between response onset (40–100 msec) and response offset. Since latency tends to increase when contrast decreases [e. g., 26], onset and offset latencies were calculated separately for each of the 6 stimulus conditions. In cells with sustained responses followed by ‘off’ responses, mean firing rate was calculated for the ‘on’ response only. Mean spontaneous activity was subtracted from mean firing rates.

Quantification of orientation tuning was achieved by fitting either a Gaussian or Von Mises formula [62] to mean firing rate vs. orientation data (Fig. 1B and 1C). The fitting procedure was implemented in Origin® software non-linear fitter. A chi-square minimization procedure was used to optimize the fit. The Von Mises equation was:
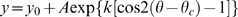



*θ* is the orientation (in rad). *y_0_* corresponds to the component of the response that lacks orientation selectivity (it does not correspond to spontaneous activity that was removed prior to fitting). *A* corresponds to the amplitude of the orientation selective response at the preferred orientation, *θ_c_*. *k* is a width factor from which the half-width at half-height (HWHH, in deg) of the tuning function can be calculated as:
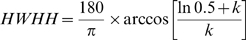



In broadly tuned cells, we found that the Von Mises fit often failed to stabilize. In these cases, the fits were made using a Gaussian curve of the form:
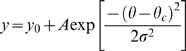
(4)


The *HWHH* was calculated as 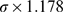.

Fits that did not stabilize even with the Gaussian function were constrained by fixing the *y_0_* value to the mean of the two lowest experimental values. We always used the same fitting equation for the different conditions in each cell. That is, if a Gaussian was used for one of the contrast/adaptation conditions, a Gaussian was also used for the other 5 conditions.

Data have been considered for further analysis only when the r^2^ of fit was >0.67. Median r^2^ were 0.935, 0.946 and 0,945 for tuning curves obtained at high, medium and low contrasts, respectively, in the mixed contrasts condition. Median r^2^ were 0.944, 0.951 and 0.949 for tuning curves obtained at high, medium and low contrasts in the constant contrast condition.

The main conclusion of this study, which is that tuning width depends on contrast, did not depend on the fit function that was used. We compared changes in half-width at half-height vs. tuning function for each pair of contrasts comparison and found that changes in tuning width did not depend on the fit function used (p>0.05, Mann Whitney U test) for 5 of the 6 contrast/adaptation conditions. The condition that showed a significant difference (p = 0.02) between fit functions corresponds to the medium vs. high contrast in the adapted situation, which was the only condition in which contrast initially had no significant effect on tuning width (see Results). For this condition, we therefore remade the paired comparison of tuning widths, splitting data for each fit function this time. When a Gauss function was used (n = 21 pairs), tuning width did not differ significantly between medium and high contrast (Wilcoxon, p = 0.1). When the von Mises function was used (n = 41 pairs), there was then a significant difference (p = 0.02), which indicated a decrease in tuning width at medium contrast compared to high contrast. Nevertheless, the median width ratio (94.97%) is very close to the median obtained when the whole sample (Gaussian+von Mises) is considered (96.01%, see Results).

#### Simple/complex cell classification

We relied on the response evoked by drifting sine-wave gratings, used for characterizing spatial frequency tuning of the cells, for classifying cells as simple or complex. PSTHs (16 bins) were computed over one cycle of the drifting grating for each spatial frequency. After subtracting the mean spontaneous activity, each histogram was Fourier-analyzed and the F0 (average firing rate) and F1 (first harmonic, response amplitude at the frequency of the grating drift) components extracted. The F1/F0 ratio, or “relative modulation” [63], was calculated for each spatial frequency. The F1/F0 value obtained with the spatial frequency that yielded the largest responses amplitude (F0 or F1) was extracted. Cells with peak amplitude <3 sp/sec were not considered. Distribution of relative modulation in our data set was bimodal, with a gap at 1. In accordance with previous studies [63], we therefore classified cells as simple when their relative modulation was >1 and complex when their relative modulation was <1.

#### Contrast-response function

This analysis has been performed on 81 single-units that showed significant responses in this protocol. PSTHs (16 bins) were computed over one cycle of the drifting grating for each contrast. After removal of spontaneous activity, each histogram was Fourier-analyzed and the F0 and F1 components extracted. Data (F0 in complex cells, F1 in simple cells) were fit using the hyperbolic ratio equation [5], [64]:
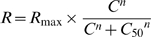
with *R_max_* corresponding to the maximal response, *C_50_* representing the contrast at with 50% of the maximal response is obtained, and the exponent *n* determining the steepness of the CRF. For cells that showed supersaturation (n = 13/81), we removed the data points that were below the maximal response for contrasts larger than the one evoking the maximal response and the fit was made on this reduced data set. For cells that did not show saturation (n = 34/81), that is, cells for which the fit provided R_max_ that would be attained at contrast >100% (and eventually, for which C_50_ would take values>100%), R_max_ was instead ascribed to the firing rate extrapolated to 100% contrast and the C_50_ was then determined from this corrected R_max_ value. These adjustments were made in order to provide a phenomenological description of the CRFs in marmoset monkey.

### Histology and electrode tract reconstruction

After completion of an electrode track, several electrolytic lesions (10 µA, 10 sec) were made at different depths through the recording microelectrode. At the end of the experiment, the animals were sacrificed with a lethal i. v. injection of sodium pentobarbitone and perfused transcardially with 0.9% saline with heparin, followed by 4% paraformaldehyde in phosphate buffer. The posterior part of the brain was removed and cryoprotection was insured by overnight immersion in 30% sucrose solution. Parasagittal sections, 40 µm thick, were cut on a freezing microtome. Sections were stained with Cresyl violet to reveal cortical layers. Recording sites positions were determined relative to electrolytic lesions positions.

### Statistics

We determined the significance of the effects of contrast and contrast adaptation for each cell individually. Since a standard error (“SE”) value was provided with each parameter of the fit, a *t* value comparing a parameter value (“V”) in two different conditions (“C1” and “C2”) could be calculated as:
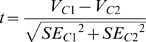
A *t* value<−2.064 indicates a significant (p<0.05; degrees of freedom 24) decrease of V_C1_ compared to V_C2_, and a *t* value>2.064 a significant (p<0.05) increase of V_C1_ compared to V_C2_.

At the population level – except when mentioned – statistical significance of differences between paired groups has been determined using the non-parametric Wilcoxon rank test. Correlations were tested using the non-parametric Spearman rank correlation test. Confidence intervals for rho were constructed using Fisher's z transformation.

## Results

### Protocol

The stimulation protocol is illustrated in Fig 1A. It was designed to tease apart the effects of contrast from those of contrast adaptation on orientation tuning, while at the same time providing the possibility to examine orientation tuning for a stimulation duration comparable to that of a visual fixation. Stimuli consisted of stationary gratings whose spatial frequency was optimal for the cells under study. Gratings were flashed for 200 msec, followed by a blank (0% contrast) lasting 200–400 msec. The orientation of the grating could take 16 different values that varied between 0 and 168.75 deg, in steps of 11.25 deg. The contrast of the grating could take three different values. These values were chosen according to CRFs analyzed on-line, and corresponded to the contrasts required to elicit approximately 20–25%, 50% and 80–90% of the maximal response. These contrasts are referred to as “low”, “medium” and “high” contrast, respectively.

During the first block of stimulation, that lasted 40–60 sec, both orientation and contrast varied randomly from one stimulus presentation to the next. This corresponds to the “mixed contrasts” block (Fig. 1A, left). In this situation, stimulus contrast changed faster than the time required for adaptation to take place. If adaptation did occur during the mixed contrasts block, this would have been for a contrast corresponding to the mean of the contrasts in use. There was therefore a *mismatch* between the contrast presented at a particular time, and the average contrast to which the neuron was adapted. This mismatch allowed us to probe the effect of contrast proper on orientation tuning, *independently of* the effect of contrast adaptation. We will refer to this condition as “mixed contrasts”. Orientation tuning curves obtained for each of the three contrasts in this mixed contrasts block are illustrated for the same cell in Fig. 1B.

In the second, third and fourth stimulation blocks, orientation still varied randomly from one stimulus presentation to the next, but the contrast was fixed to one value at a time for each block: either low, medium or high (Fig. 1A). Each block duration was 20 sec at least, so as to allow contrast adaptation to take place. For the cell shown in Fig. 1, firing rate adaptation can be seen in the PSTH (portions above medium and high contrast) as a decline in firing rate as a function of time. Firing rate decay was fit with a single exponential (black line). Once a steady state of firing was achieved (assumed to begin at a time corresponding to 3 times the adaptation time constant), mean firing rate for each contrast and orientation was extracted and used to calculate orientation-tuning curves (Fig. 1C). This corresponds to a situation in which the cell has adapted to a contrast that *matched* with the one used to stimulate the cell. This allowed us to examine the effect of contrast on orientation selectivity, *including* the effect of contrast adaptation. This condition is referred to as “constant contrast” condition.

The present study is based on extracellular recordings that have been performed in area V1 of 6 marmoset monkeys. The sample consists of 114 cells that responded to at least 1 of the 6 orientation vs. contrast conditions. Eighty-seven of these cells (76%) were orientation selective, a proportion very similar to that reported in previous studies of marmoset V1 [46], [47].

### Adaptation to constant contrast and time constant of contrast adaptation

To determine the number of cells that showed significant contrast adaptation, we compared for each single cell the number of spikes, averaged across all orientations, in the first 5 sec of the response to constant contrast presentation with the number of spikes counted between 15 and 20 sec (see Methods). When considering all cells, whether orientation-selective or not, that gave a significant response in at least one of the constant contrast blocks, we found that 9/69 cells (13.0%) adapted with low contrast stimuli, 54/105 cells (51.4%) adapted with medium contrast stimuli, and 69/109 cells (63.3%) adapted with high contrast stimuli.

**Figure 2:**
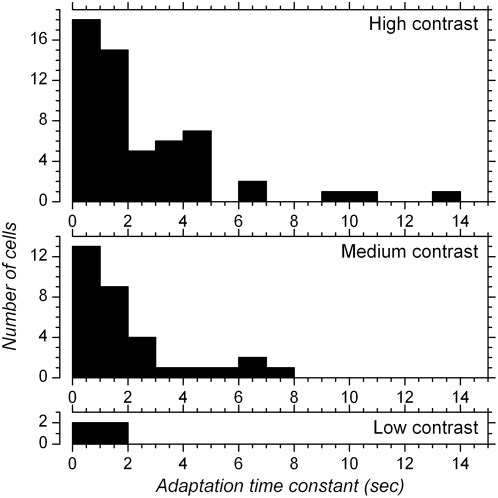
Distribution histograms of contrast adaptation time constants. Adaptation time constants were determined from single exponential curves fitted to PSTHs obtained with high (upper histogram), medium (middle histogram) and low (lower histogram) contrasts in constant contrast blocks, as exemplified in Fig. 1A.

Time constant of contrast adaptation was estimated using exponential fits made to PSTHs with bin width of 0.8–1.2 sec (Fig. 1A). Adaptation time constant could not be determined in all cases because of the noisiness in the PSTHs resulting from randomly varying orientations (low contrast: 5 cases; medium contrast: 22 cases; high contrast: 7 cases; see Methods). Distribution histograms of adaptation time constants for each of the three contrasts levels are presented in Fig. 2. Median time constant of adaptation was 0.6 sec for low contrast stimuli (n = 4), 1.2 sec for medium contrast (n = 32) and 1.6 sec for high contrast stimuli (n = 56) (due to the skewness in the distributions the means were higher: 0.6, 1.9 and 2.5 sec respectively). Approximately 15% of the cells showed adaptation time constant <0.5 second. Only a few cells (15% at medium contrast and 20% at high contrast) showed adaptation time constant equal to, or larger than, 4 sec. On average, the time constants we report here are shorter than those obtained with drifting stimuli [31]–[37] but the presence of short time constants is consistent with results obtained with stationary stimuli [65], [66].

### Examples of orientation tuning at different contrasts, mixed and constant contrasts conditions

A first example illustrating the effects of contrast on orientation tuning when the contrast presented at a particular time, and the average contrast to which the neuron was adapted, did not correspond (mixed contrasts) is depicted in Fig. 1B. Orientation tuning data were fit with the von Mises equation (Methods). Reducing the contrast of the stimulus reduced, as expected, the amplitude of the response. In addition, there was a noticeable change in the width of the tuning curves: the HWHH with the lowest contrast was 12.2 deg while it was 19.0 deg with the highest contrast (changes in width can be appreciated in the inset of Fig. 1B, where tuning curves have been normalized to same amplitude and preferred orientation). This difference was significant (t = −5.849, p<0.05; see Methods). Tuning width was also significantly different between medium (16.8 deg) and low contrast (t = −4.659) and between high and medium contrast (t = −2.687). For this cell in this stimulation regime, orientation tuning does not appear to be contrast-invariant. One can also notice that the baseline response is slightly elevated for the high contrast response, at about 2 sp/sec, compared to the low and medium contrast, where the baseline is near 0 sp/sec.

Fig. 1C illustrates the tuning curves obtained when the contrast used to stimulate the cell matched with the contrast to which the cell were adapted. Comparing Fig. 1C to Fig. 1B shows some of the changes brought to orientation tuning curves by contrast adaptation. In this cell, amplitude of responses to high contrast stimuli were not very different between mixed and constant contrast condition, and HWHHs also were very similar (19.2 vs. 19 deg). On the other hand, with low contrast stimuli, response amplitude was higher in the constant contrast condition (Fig. 1C), and the HWHH was broader compared to that in the mixed contrasts condition (15.4 vs. 12.2 deg). With constant contrast, tuning width did not differ significantly anymore between medium and high contrast (t = −1.462) and between low and medium contrast (t = −1.714), but was still significantly different between low and high contrast (t = −2.504).

**Figure 3:**
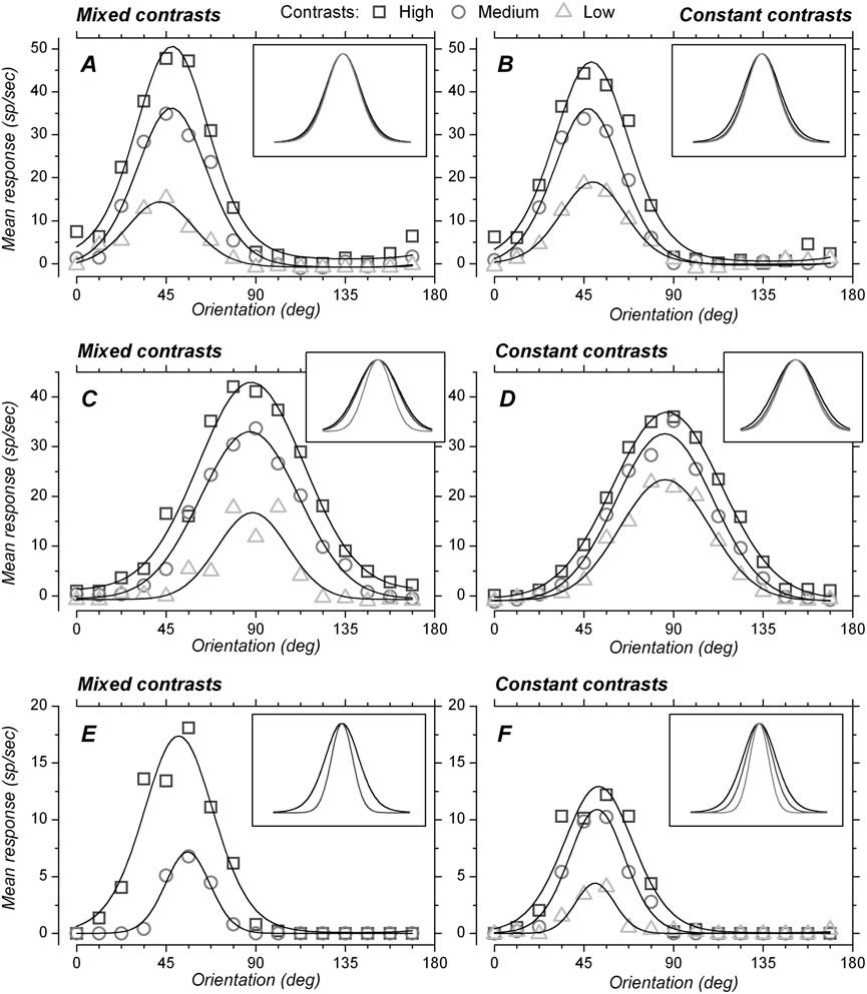
Orientation tuning with different contrasts in mixed and constant contrast blocks, additional examples. Symbols represent the mean firing rate for each orientation and contrast, and the lines correspond to the von Mises (A, B, E, F) or Gauss (C, D) equations fitted to the orientation-response data. Inset shows fitted lines normalized to the same preferred orientation and to the same height, to facilitate comparison of tuning widths. A. For this cell, contrast, in the mixed contrasts condition, had little effect on orientation tuning width, although response amplitude depended strongly on contrast. Contrasts were 11.3, 16 and 22.6%. HWHH were 20.1, 21.1 and 21.9 deg for low, medium and high contrasts, respectively. B. For the same cell, orientation tuning width was also little affected by contrast in the constant contrast blocks. HWHH were 19.3, 20.2 and 22.4 deg for low, medium and high contrasts, respectively. C. This cell showed, in the mixed contrasts condition, reduced tuning width with low contrast stimuli compared to high or medium contrast stimuli. Contrasts were 22.6, 32 and 64%. HWHH were 20.7, 29.2 and 31.2 deg for low, medium and high contrasts, respectively. D. After matched adaptation (constant contrast), the range of HWHH appears to be less wide. HWHH were 26.7, 28.7 and 32.1 deg for low, medium and high contrasts, respectively. E. No significant response was obtained in this cell with low contrast stimuli (35%) in the mixed contrasts block. The tuning curve obtained with high contrast (90%) was broader (HWHH: 20.9 deg) than the tuning curve obtained with medium contrast (50%, HWHH: 13.2 deg). F. Despite adaptation to matched contrasts, the same cell shows differences in HWHH between low (11.6 deg), medium (16.6 deg) and high (20.85 deg) contrasts.

Additional examples are presented in Fig. 3. Fig. 3A shows a cell for which decreasing contrast, in the mixed contrasts condition, did not induce significant change in tuning width (p>0.05), whatever the contrast comparison (Fig. 3A, inset). This corresponds to a cell for which orientation tuning was contrast-invariant. Compared to the mixed contrasts condition, the constant contrast condition changed the response amplitude (Fig. 3B, lower response to high contrast, and higher response to low contrast). However, tuning width remained very similar for the different contrasts (p>0.05). Contrast-invariance of tuning width, already present in the mixed contrasts protocol (Fig. 3A), was still present in the constant contrast condition.

For the cell of Fig. 3C, tuning width in the mixed contrasts condition did not differ significantly between medium and high contrast (t = −0.988) but was significantly narrower at low contrast compared to medium and high contrast (t = −2.509 and −2.853, respectively). For the same cell in the constant contrasts condition (Fig. 3D), tuning width at low contrast was still significantly less than at high contrast (t = −3.789), but did not differ significantly from that obtained at medium contrast anymore (t = −1.188). In this cell therefore, orientation tuning appeared to be less affected by contrast in the constant contrast condition. It can also be seen that the response to low contrast in the constant contrast condition (Fig. 3D) was larger than the response to low contrast in the mixed contrasts condition (Fig. 3C). The response to high contrast showed the opposite pattern.

The example in Fig. 3E–F shows that some cells still showed strong effects of contrast on tuning width, despite adaptation to each of the contrasts individually. Note that, in this cell, response to low contrast was significant in the constant contrast condition (Fig. 3F), but was not significant in the mixed contrasts condition (Fig. 3E). The tuning width obtained at medium contrast appears significantly narrower than at high contrast (t = −5.966) in the mixed contrasts as well as in the constant contrast condition (t = −3.021). In this last condition, the tuning width at low contrast was significantly less than at high and medium contrast (t = −4.319 and −3.271, respectively).

We next examined, at the population level, how the orientation tuning parameters were modified by contrast and contrast adaptation.

### Effect of contrast on tuned response amplitude

**Figure 4:**
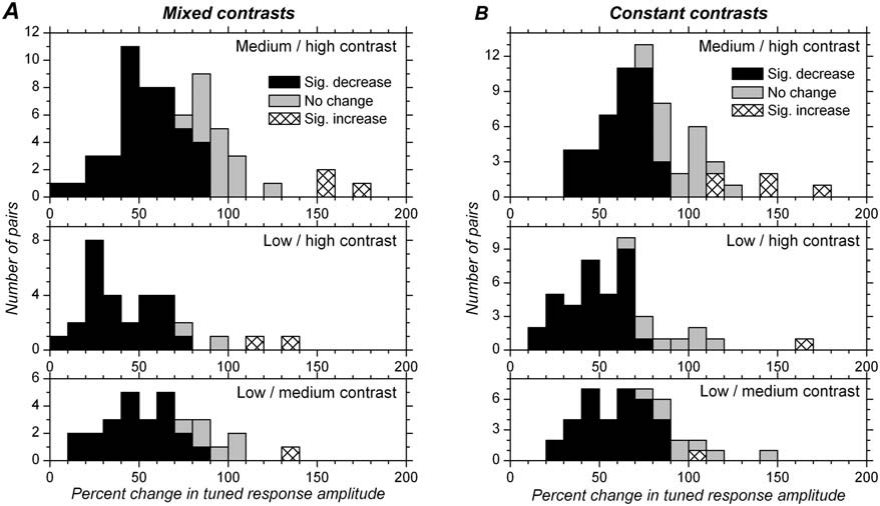
Distribution of changes in tuned response amplitude with different contrasts. A. Mixed contrasts. B. Constant contrasts. Tuned response amplitude for medium contrast is expressed as a percentage of the tuned response amplitude at high contrast in the upper histograms. Tuned response amplitude for low contrast is expressed as a percentage of the tuned response amplitude at high contrast in the middle histograms, and as a percentage of the tuned response amplitude at medium contrast in the lower histograms. 100% on *x*-axis corresponds to no change in response amplitude. Relative to 100%, all distributions are shifted to the left, indicating decreased response strength with decreased contrast in nearly all cases. Black bars correspond to significant decreases, and hatched bars to significant increases in response amplitude, tested at the single cell level (p<0.05, t test). In the vast majority of cells, the response amplitude was significantly lower when contrast was decreased.

We first examined changes in response amplitude resulting from changes in stimulus contrast. Response amplitude refers here to parameter “*A*” in the fitting equations, which represents the amplitude of the orientation-tuned component in the neuronal responses (changes for the parameter “*y_0_*”, representing the amplitude of the untuned component in the response, will be presented later). Fig. 4 shows, as distribution histograms, percent change in tuned response amplitude for cells in which responses at two or three contrasts could be compared.

In the mixed contrasts condition, tuned response amplitude varied with contrast, and, not unexpectedly, firing rates were lower at low contrast compared to higher contrasts in the vast majority of the cells (Fig. 4A). We examined the significance of the changes at the single cell level (t test, see Methods). The black bars (Fig. 4A) represent the cells for which response amplitude was significantly decreased (p<0.05) when contrast was decreased. This occurred in 71% (44/62) of the cells when comparing medium and high contrast, and in 86.7% (26/30) of the cells when comparing low and high contrast. At the population level, median firing rate was 14.2 sp/sec at high contrast, 9.7 sp/sec at medium contrast, and 7.5 sp/sec at low contrast (Table 1). Differences were highly significant (p<0.0001 for medium vs. high, low vs. high and low vs. medium contrasts in paired comparisons). The tuned response amplitude at medium contrast represented 64.4% (median) of that obtained at high contrast. The tuned response amplitude at low contrast represented 39.5% of that obtained at high contrast and 57.2% of that obtained at medium contrast (Table 2).

**Table 1:** Tuned response amplitude, half-width at half-height and relative untuned response amplitude at different contrasts, in mixed or constant contrasts conditions.

Mixed contrasts (mismatched adaptation)			
*Contrast*	Low	Medium	High
	n = 31	n = 67	n = 70
*Tuned response amplitude (sp/sec)*	7.5 [10.2]	9.7 [11.2]	14.2 [19.4]
	9.1±7.1	11.8±9.6	18.1±13.6
*Half-width at half-height (deg)*	12.2 [9.8]	18.9 [13.6]	23.1 [14.8]
	13.9±6.2	20.6±9.6	23.8±9.7
*Relative unselective response amplitude (%)*	0.0 [4.4]	1.4 [5.0]	3.2 [8.9]
	0.7±4.1	4.6±12.4	6.8±10.6

Constant contrasts (matched adaptation)			
*Contrast*	Low	Medium	High
	n = 46	n = 63	n = 80
*Tuned response amplitude (sp/sec)*	7.7 [14.4]	10.1 [11.8]	10.5 [16.1]
	10.4±8.3	12.9±9.9	14.8±12.2
*Half-width at half-height (deg)*	15.5 [10.9]	20.5 [15.7]	21.8 [10.7]
	18.4±9.4	21.9±9.4	23.1±8.3
*Relative unselective response amplitude (%)*	0.6 [3.7]	0.6 [4.5]	3.1 [10.9]
	3.0±9.8	1.4±8.6	7.5±12.3

For each column, “n” indicates the number of cells with significant response and acceptable orientation tuning fit (see methods). For each parameter, the numbers on the top row are the median and the interquartile (between brackets). The numbers on the bottom row correspond to the mean±1 standard deviation.

**Table 2:** Paired comparison and associated ratios for tuned response amplitude and half-width at half-height, and differences for relative untuned response amplitude, at different contrasts and with matched or mismatched adaptation.

Mixed contrasts (mismatched adaptation)			
*Contrast comparison*	Low vs. High	Low vs. Medium	Medium vs. High
	n = 30	n = 30	n = 62
*Tuned response amplitude (ratio, %)*	39.6 [37.1]	57.2 [34.2]	64.4 [35.9]
	47.4±29.8	60.0±28.0	68.8±31.9
	(p<0.0001)	(p<0.0001)	(p<0.0001)
*Half-width at half-height (ratio, %)*	73.9 [35.5]	76.6 [35.0]	88.9 [25.2]
	72.6±27.9	76.2±22.0	86.6±22.4
	(p<0.0001)	(p<0.0001)	(p<0.0001)
*Relative unselective response amplitude (difference, %)*	0.36 [5.20]	−0.78 [4.62]	1.22 [5.06]
	1.49±5.38	−0.85±4.56	2.32±5.28
	(ns)	(ns)	(p = 0.0035)

Constant contrasts (matched adaptation)			
*Contrast comparison*	Low vs. High	Low vs. Medium	Medium vs. High
	n = 43	n = 43	n = 62
*Tuned response amplitude (ratio, %)*	54.9 [27.9]	67.2 [33.1]	74.0 [28.9]
	58.1±29.5	67.1±25.1	78.5±27.7
	(p<0.0001)	(p<0.0001)	(p<0.0001)
*Half-width at half-height (ratio, %)*	84.5 [29.0]	89.5 [20.7]	96.0 [23.8]
	86.6±21.9	89.9±19.2	100.6±19.5
	(p = 0.0001)	(p = 0.0002)	(ns)
*Relative unselective response amplitude (difference, %)*	1.28 [5.43]	0.06 [1.62]	1.25 [5.32]
	3.10±5.23	0.74±4.74	2.48±5.93
	(p = 0.0006)	(ns)	(p = 0.0008)

For each column, “n” indicates the number of pairs of cells that were compared. For each parameter comparison, the numbers on the top row are the median and the interquartile (between brackets). The numbers on the middle row correspond to the mean±1 standard deviation. The lower row shows *p* values in paired comparisons (ns: not significant). For the tuned response amplitude and the half-width at half-height, numbers correspond to the ratios (in percent) of values for one contrast vs. the other: low/high, low/medium and medium/low contrast. For the relative unselective response amplitude, numbers correspond to the difference of values for one contrast vs. the other: high minus low, medium minus low, and high minus medium.

The amplitude of the tuned response component decreased with decreases in contrast in the adapted situation as well (Table 1), but differences were less than in the mixed contrasts condition. Percent changes in tuned response amplitude are presented in Fig. 4B and Table 2. At the single cell level, response amplitude significantly decreased in 64.5%, 79.1% and 79.1% of the cells when comparing medium and high contrast, low and high contrast, and low and medium contrast, respectively. At the population level, responses obtained at low contrast were significantly lower than at medium contrast (p<0.0001), and responses at medium contrast were significantly lower than at high contrast (p<0.0001). Tuned response amplitude at low contrast represented 67.2% (median) of the amplitude obtained at medium contrast, and tuned response amplitude at medium contrast represented 78.5% of the amplitude obtained at high contrast. However, as a result of adaptation to each contrast individually, differences in tuned response amplitude are of lesser importance than those obtained in the mixed contrasts condition (57.2% and 64.4% respectively, Fig. 4A).

### Effect of adaptation on tuned response amplitude

During the mixed contrasts block, neurons adapted to a contrast representing the mean of the three contrasts used, whereas they adapted to the only contrast in use during the constant contrast blocks. It was therefore expected that response should be lower for high contrast after adaptation to high contrast than after adaptation to the mixed contrasts. Conversely, it was expected that responses should be *higher* for low contrast after adaptation to low contrast than after adaptation to mixed contrasts. Finally, given that, by experimental design, medium contrast should have yielded a response close to the mean obtained with the mean of the three contrasts in use, response amplitude after adaptation to medium contrast was expected not to differ much from response amplitude after mixed contrasts adaptation.

**Figure 5:**
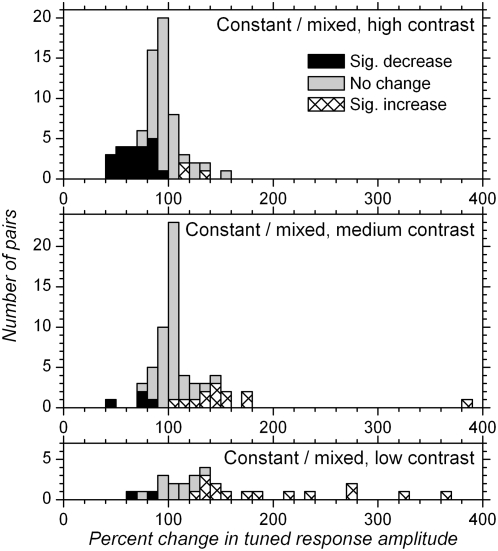
Comparison of tuned response amplitude with the same contrast in mixed and in constant contrast blocks. The distribution histograms show response amplitude obtained, for a given contrast, in constant contrast blocks as a percentage of that obtained in the mixed contrasts block. Upper histogram shows distribution for high contrast, middle histogram for medium contrast, and lower histogram for low contrast. Bar filling refers to significance of changes at the single cell level (t-test): black indicates significant decrease of response amplitude after matched adaptation compared to unmatched adaptation, gray indicates lack of significant changes (p>0.05), and hatched indicates significant increase. 100% on x-axis corresponds to no change in response amplitude. Relative to 100%, the distribution for high contrast is shifted to the left, indicating that response amplitude was larger for high contrast when neurons were adapted to a mixture of contrasts, compared to when neurons were adapted to the high contrast. On the contrary, the distribution of percent change for low contrast stimuli appears shifted to the right: response amplitude was lower on average for low contrast stimuli when neurons were adapted to mixed contrasts compared to when neurons were adapted to the low contrast. The distribution for medium contrast is more centered, although responses to medium contrast were slightly stronger, on average, after adaptation to medium contrast compared to adaptation to mixed contrasts.

The results obtained were close to this expectation. Changes in response amplitude were calculated as the ratio of the response amplitude, for a given contrast, after adaptation to that contrast in the constant (cst) contrast block, to the response amplitude to the same contrast in the mixed (mix) contrasts block: 100×*A*_cst_/*A*_mix_. Fig. 5 plots distribution histograms for this ratio. At the single cell level, with high contrast stimuli, matched adaptation (constant high contrast) resulted in a significant decrease in response amplitude in 30.4% (Fig. 5, top histogram, black bars) of the cells and no significant change in 65.2% of the cells, compared to unmatched adaptation. With medium contrast, matched adaptation induced a significant increase in response amplitude in 21.3% of the cells (Fig. 5, middle histogram, hatched bars), and no significant change in 72.1%. Finally, with low contrast stimuli, matched adaptation induced an increase in response amplitude in 53.6% (Fig. 5, bottom histogram, hatched bars), and no significant change in 39.3% of the cells, compared to unmatched adaptation.

At the population level, response amplitude with high contrast stimuli was significantly (p<0.0001) lower after adaptation to high contrast compared to adaptation to mixed contrasts (median percent change: 91.3%). Response amplitude with medium contrast was significantly (p = 0.006) larger after adaptation to medium contrast than in mixed contrasts blocks but the median (102.6%) indicates relatively small changes in this case. Finally, response amplitude with low contrast stimuli was significantly (p = 0.0004) *higher* after adaptation to low contrast than in mixed contrasts blocks (median 132.9%); in this later case, responses to low contrast stimuli were relatively depressed due to adaptation to the mean of the three contrasts in use in the mixed contrasts blocks, and recovered from this depression when given time to adapt to the low contrast only.

### Effect of contrast on preferred orientation

**Figure 6:**
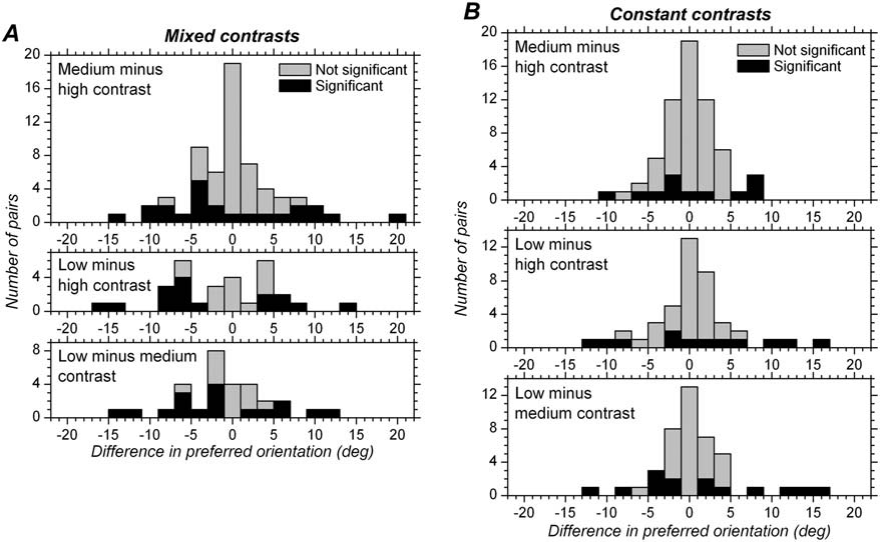
Distribution of differences in preferred orientation with different contrasts. A. Mixed contrasts. B. Constant contrasts. Upper histograms show the differences for medium vs. high contrast. Middle histograms show the differences for low vs. high contrast. Lower histograms show the differences for low vs. medium contrast. Black bars correspond to cases for which the difference was found to be significant at the single cell level (p<0.05, t test).

We next examined whether changing contrast modified neurons preferred orientation, as has been reported in a cat study [67]. We quantified changes in preferred orientation (*θ_c_*) by calculating the difference between the value obtained with one contrast (C1) from the one obtained with a higher contrast (C2), *θ_cC1_*−*θ_cC2_*. Distributions of differences in preferred orientation are shown in Fig. 6 for each of the 3 pairs of comparison.

In the mixed contrasts condition, a significant change in preferred orientation occurred in 37.1%, 53.3% and 56.7% of the cells for high vs. medium, high vs. low and medium vs. low contrasts, respectively (Fig. 6A). With adaptation to each single contrast (Fig. 6B), significant changes in preferred orientation occurred less often than in the mixed contrasts blocks: 19.4% for high vs. medium contrast, 27.9% for high vs. low contrast, and 32.6% for medium vs. low contrast. This data show that preferred orientation does change significantly in a considerable fraction of the cells when the contrast is modified, especially in the mixed contrasts condition.

**Figure 7:**
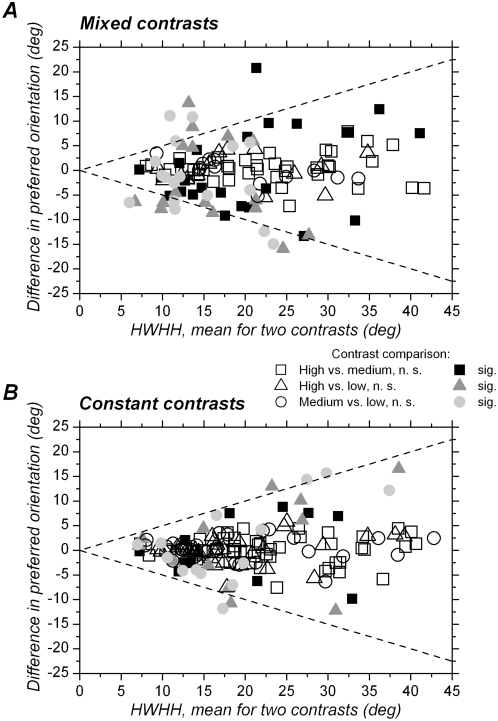
Changes in preferred orientation vs. half-width at half-height of tuning curves. A. Mixed contrasts. B. Constant contrasts. The *x*-axis corresponds to the mean of the two HWHHs obtained with the two contrasts that are compared. The *y*-axis represents the difference in preferred orientation observed with the same contrasts. Squares: high compared to medium contrast. Triangles: high compared to low contrast. Circles: Medium compared to low contrast. Cells for which preferred orientation changed significantly (p<0.05, t test) are represented by filled symbols (“sig”) and cells for which preferred orientation did not change significantly by open symbols (“n. s.”). The diagonals represent the relation *y* = ±0.5*x*. Data points between the diagonals correspond to cells for which the difference in preferred orientation is less than the mean HWHH.

However, the differences in preferred orientation appear to be small in most cases. At the population level, the absolute values of preferred orientation differences were <8 deg for 80% of the cells in the mixed contrasts condition, and <6 deg in 80% of the cases in the constant contrast condition. Furthermore, as shown in Fig. 7, the changes in preferred orientation were generally less than the tuning width of the cells. The scatter plots (Fig. 7) show the difference in preferred orientation against the mean of the HWHH obtained for the same contrasts comparison. The diagonal lines correspond to a change in preferred orientation equal to ± half of the mean HWHH. The majority of data points are confined between these lines. The scatter increases with increase in tuning width; in other words, changes in preferred orientation may be large for broadly tuned cells but are in general small for sharply tuned cells. Cells whose preferred orientation changed by a value larger than the mean HWHH are relatively few in the mixed contrasts condition (Fig. 7A) and even rarer in the constant contrast condition (Fig. 7B).

### Effect of contrast in the mixed contrasts condition on the half-width at half-height of orientation tuning curves

**Figure 8:**
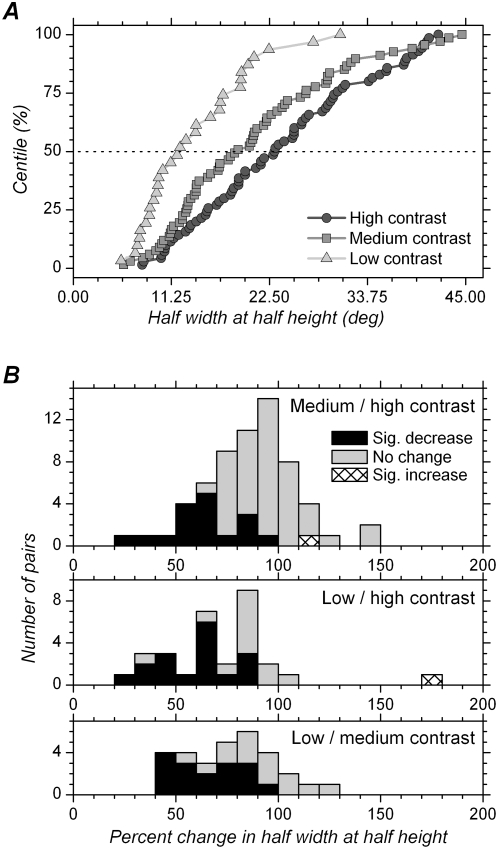
Changes in orientation tuning width with different contrasts in mixed contrasts blocks. A. Cumulative distribution of HWHH for the three different contrasts. Horizontal dashed line corresponds to the median. B. Distribution of percent change in HWHH with different contrasts. HWHH for medium contrast is expressed as a percentage of the HWHH at high contrast in the upper histogram. HWHH for low contrast is expressed as a percentage of the HWHH at high contrast in the middle histogram, and as a percentage of the HWHH at medium contrast in the lower histogram. 100% on *x*-axis corresponds to no change in HWHH. Relative to 100%, all distributions are shifted to the left, indicating decreased HWHH with decreased contrast. Black bars in histograms correspond to significant decrease in HWHH when contrast decreases, and hatched bars to significant increase in HWHH, tested at the single cell level (p<0.05, t test).

We found that, in the mixed contrasts condition, contrast had a strong and highly significant effect on the HWHH of orientation tuning curves, with HWHHs being on average larger with higher contrast. Cumulative distributions of HWHHs for each contrast group are shown in Fig. 8A. Means and medians are presented in Table 1. Distributions obtained for medium and high contrast are comparable to those obtained using flashing stimuli in behaving macaques [68]. Median HWHH was 23.1 deg with high contrast (n = 70), similar to values previously reported in marmoset V1 [46], [48]. It was less (18.9 deg) at medium contrast (n = 67). The cumulative distribution for HWHH at low contrasts is clearly displaced to the left, and the median HWHH at low contrast was only 12.2 deg (n = 31).

Distributions of percent change in HWHH are presented in Fig. 8B and means and medians displayed in Table 2. Changes are expressed as the HWHH for a given contrast (low or medium) as a percentage of the HWHH obtained with higher contrasts (medium or high). Cells for which tuning width decreased significantly at the single cell level are indicated by black bars. Significant decrease in tuning width was observed in 27.4% (17/62) of the cells when comparing medium and high contrast, in 56.7% (17/30) of the cells when comparing low and high contrast, and in 53.3% (16/30) of the cells when comparing low and medium contrast. These proportions are much larger than the expected proportion of false positives (type 1 error, <5%), given our threshold criteria for significant differences (p<0.05, Methods).

Given that the sample size varied for the different contrasts, we used paired comparisons to compare data at the population level. We found that HWHH at medium contrast was significantly less than at high contrast (p<0.0001, n = 62 pairs), with the HWHH at medium contrast representing 88.9% (median) of the HWHHs at high contrast. Similarly, HWHH at low contrast was significantly less than at high contrast (p<0.0001, n = 30 pairs), with the HWHH at low contrast representing 73.9% of the value obtained at high contrast. Finally, HWHH at low contrast was significantly less than at medium contrast (p<0.0001, n = 30 pairs), and the median value of percent change indicate that the HWHH at low contrast represented 76.4% of that obtained at medium contrast (Table 2).

### Effect of contrast in constant contrast conditions on the half-width at half-height of orientation tuning curve

**Figure 9:**
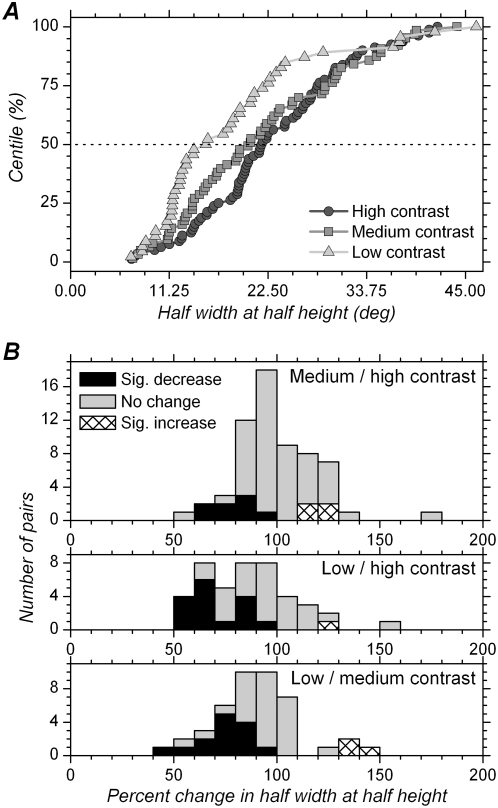
Changes in orientation tuning width with different contrasts for constant contrast blocks. A. Cumulative distribution of HWHH for the three different contrasts. B. Distribution of percent change in HWHH with different contrasts. Same conventions as in Figure 8. Both cumulative distributions and percent change distributions show reduced effect of contrast on tuning width in the constant contrast conditions compared to the mixed contrasts condition (Fig. 8).

In contrast to what was observed in the mixed contrasts condition, the cumulative distributions (Fig. 9A) show that HWHHs of orientation tuning curves obtained in the constant contrast condition with high and medium contrast are quite similar. However, HWHHs for tuning curves obtained at low contrast still seem to be narrower than those obtained at medium and high contrasts, despite the fact these data were obtained once the cells were adapted to each of the contrasts in use (Table 1). These impressions are statistically confirmed in paired comparisons (Table 2). HWHH at medium contrast was not significantly different from that obtained at high contrast (p = 0.47, n = 62 pairs), with the HWHH at medium contrast representing 96.0% (median) or 100.6% (mean) of the HWHH at high contrast (Fig. 9B). At the single cell level, tuning width was not significantly modified in 80.6% of the cells, while 6.5% showed significant increase in tuning width, and 12.9% significant decrease in tuning width.

On the other hand, HWHHs at low contrast were still significantly less than at high contrast (p = 0.0001, n = 43 pairs) and still significantly less than at medium contrast (p = 0.0002, n = 43 pairs). HWHH at low contrast represented 84.5% of the value obtained at high contrast and 89.5% of that obtained at medium contrast (Fig. 9B). However, although still significant, these differences are less than those obtained in mixed contrasts conditions (73.9% and 76.4%, respectively, Fig. 8). At the single cell level, the proportion of cells showing a significant decrease in tuning width also appears to be less than in the mixed contrasts condition: it is 37.2% for low vs. high contrast, and 32.6% for low vs. medium contrast, compared to 56.7% and 53.3% in the mixed contrasts condition (Fig. 8). Contrast adaptation therefore restored invariance of orientation tuning when comparing high and medium contrast, and reduced differences in tuning width when comparing with low contrast.

### Interaction between change in response strength and change in tuning width

**Figure 10:**
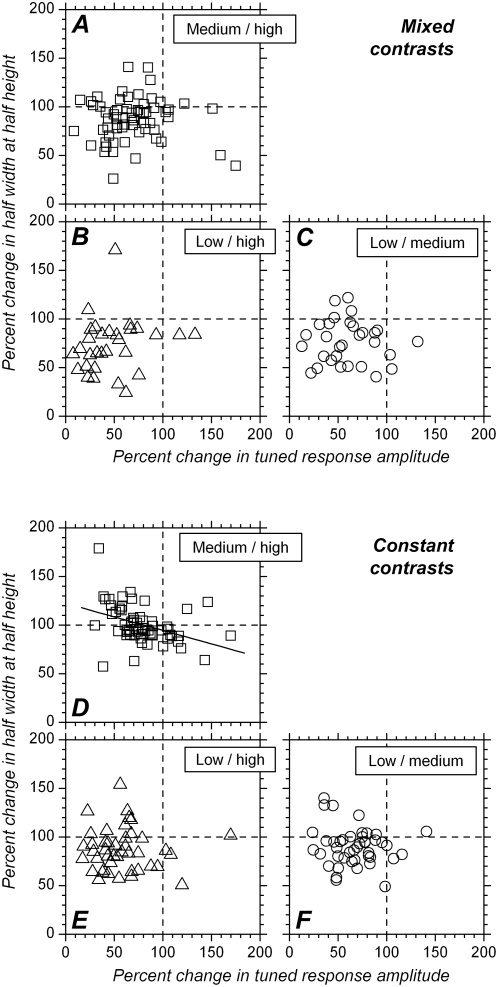
Percent change in orientation tuning width vs. percent change in tuned response amplitude. In these scatter plots, the *x*-axis represents percent change in tuned response amplitude and the *y*-axis represents percent change in HWHH. A. Medium vs. high contrast, mixed contrasts condition. B. Low vs. high contrast, mixed contrasts condition. C. Low vs. medium contrast, mixed contrasts condition. In these three scatter plots, most data points are located in the quadrant delimited by 0 and 100% on both x and y axis, indicating that most cells showed both reduced response amplitude and reduced HWHH when contrast was decreased. However, the two variables were not significantly correlated. D. Medium vs. high contrast, constant contrast condition. E. Low vs. high contrast, constant contrast condition. F. Low vs. medium contrast, constant contrast condition. In the scatter plots in E and F, most data points can be found in the quadrant delimited by 0 and 100% on both x and y axes, indicating that most cells showed both reduced response amplitude and reduced HWHH when contrast was decreased. This is not the case for the scatter plot in D, reflecting the fact that orientation tuning width was not different, on average, between medium and high contrast after adaptation. There is, however, a significant inverse relationship between the two variables in this case. The line corresponds to the linear relationship between the two variables.

Decreasing contrast reduced both tuning width and response strength. We next examined interactions between these two parameters. Indeed, a simple “iceberg” effect predicts that orientation tuning becomes broader when response becomes stronger. This, however, was not found to be the case. The scatter plots in Fig. 10 show percent changes in HWHH plotted against percent changes in tuned response amplitude. For what concerns the mixed contrasts condition (Fig. 10A–C), most data points can be found in the quadrant defined by the 0–100% ranges on both the x and y axes, indicating that most cells showed both lower response amplitude and sharper tuning at lower contrast. However, there was no significant correlation between changes in tuning width and change in response strength (for medium vs. high contrast changes: Rho = 0.12, p = 0.34, 95% confidence interval: −0.13 to 0.38; for low vs. high contrast changes: Rho = 0.25, p = 0.17, 95% confidence interval: −0.12 to 0.63; for low vs. medium contrast changes: Rho = −0.008, p = 0.97, 95% confidence interval: −0.39 to 0.37).

No significant correlation was obtained between changes in tuning width and changes in response strength for low vs. high contrast and for low vs. medium contrast in the constant contrast condition as well (Fig. 10E and 10F, respectively) (low vs. high: Rho = −0.015, p = 0.9, 95% confidence interval: −0.32 to 0.29, low vs. medium: Rho = −0.082, p = 0.6, 95% confidence interval: −0.39 to 0.23). However, although HWHH at high and medium contrasts do not differ significantly at the population level after adaptation, there nevertheless appears to be an interaction between changes in response strength and changes in HWHH, which indicates there is still a remnant effect of contrast on orientation selectivity for these two contrasts. This is illustrated in Fig. 10D, where a significant correlation appears between change in response strength and change in tuning width for medium vs. high contrast (Rho = −0.492, p = 0.0001, 95% confidence interval: −0.79 to −0.28). Nevertheless, the trend reported here is relatively weak (r^2^ = 0.156 with a linear relationship). It is, furthermore, a negative correlation, suggesting *increases* in tuning width at medium contrast, provided response amplitude is less than at high contrast.

The distribution histograms (Fig. 4) and scatter plots (Fig. 10) also show that the firing rate in a small number of cells either did not change significantly when contrast decreased, or was actually significantly higher at medium, and sometimes at low contrast than at high contrast. This corresponds to cells that were saturating and “supersaturating”, respectively. (Note that presence of saturating or supersaturating cells was initially not desired. It is to be remembered that, during the course of the experiment, the 3 contrast values to be used for the stationary flashing gratings were chosen on the basis of CRFs generated with drifting gratings and analyzing the multi-unit response. Single-unit isolation was made off-line, and it sometimes happened that one isolated single-unit had lower contrast sensitivity and saturated at lower contrasts in comparison to the multi-unit. Furthermore, it also sometimes happened that, for a given single-unit, response amplitude obtained with stationary stimuli at different contrasts differed from that expected from the CRFs obtained with drifting gratings.) However, these neurons (in particular Fig. 10A) show that the HWHH could decrease even when response amplitude was larger with lower contrasts.

The fact that there is no statistically significant correlation between change in response strength and change in tuning width in 5/6 cases and a negative correlation in the remaining, and the fact that, for some of the saturating and supersaturating neurons, tuning was broader with higher contrast, suggest that the effect of contrast on tuning width is largely independent of the effect of contrast on response amplitude.

### Comparison of tuning width for one given contrast, with and without matched adaptation

The data presented in Fig. 8 and 9 compared tuning widths obtained with different contrasts in the same stimulus regime: either after adaptation to the same contrast, or with adaptation to a mixture of contrasts whose average does not correspond to at least 2 of the 3 contrasts used for calculating the tuning curves. We shall now examine changes in tuning width obtained for the same contrast, but in different adaptation regimes. This directly examines the effects of contrast adaptation on orientation tuning.

**Figure 11:**
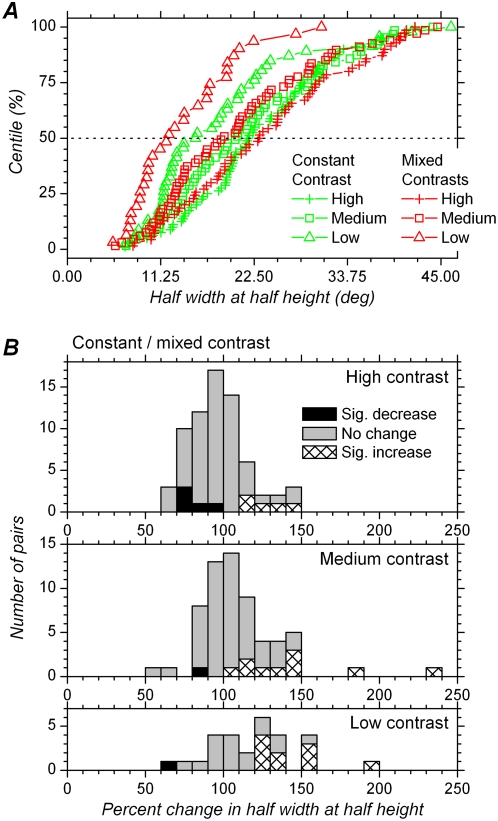
The effects of contrast and contrast adaptation on HWHH. A. Cumulative distribution of HWHH. Data in red were obtained with the mixed contrasts block and data in green obtained with constant contrast blocks. The effect of matched contrast adaptation is a narrowing of the distributions, with the largest shift observed for the low contrast. B. Distributions of percent change in HWHH for one contrast in two stimulation regimes. HWHH obtained in the constant contrast block for a given contrast (matched adaptation) is expressed as a percentage of the one obtained for the same contrast in the mixed contrasts block (mismatched adaptation). Cells showing significant (t-test, p<0.05) decrease in HWHH with adaptation to constant contrast are indicated in black, and cells showing significant increase in HWHH by hachure. Upper histogram: adaptation to high contrast resulted in a small but significant reduction of HWHH compared to adaptation to mixed contrasts (median: 96.3%). Middle histogram: adaptation to medium contrast resulted in a small but significant increase in HWHH compared to adapting to mixed contrasts (median: 105.0%). Lower histogram: adaptation to low contrast resulted in a significant and larger increase in HWHH compared to adapting to mixed contrasts (median: 120.7%).

The consequence of adaptation to each contrast individually is a narrowing of the distributions of HWHHs, as shown in Fig. 11A (this combines data from Fig. 8A and Fig. 9A). Data in red correspond to data obtained in the mixed contrasts blocks. Data in green correspond to data obtained after adaptation to the contrast used to make the measurements (constant contrast conditions). The distribution for low contrast (triangles) is clearly shifted to the right by adaptation to low contrast. This corresponds to a broadening of the tuning curves by adaptation to low contrast, compared to adaptation to mixed contrasts. When examined in a paired fashion the difference is highly significant (p = 0.004). Percent changes in HWHH (Fig. 11B, bottom) were calculated as the HWHH obtained in constant contrast blocks divided by HWHH obtained in mixed contrasts block. It shows a median of 120.7% (n = 28). When examined at the single cell level, significant increase in tuning width was observed in 35.7% of the cells (10/28, hatched bars).

HWHHs obtained with high contrast stimuli showed the opposite trend, although in a less striking fashion: a narrowing of the tuning curves after adaptation to the high contrast, compared to adaptation to the mixed contrasts. A small proportion of cells showed significant increase and decrease in tuning width (7.2%, 5/69 cells in both cases, Fig. 11B, top histogram). At the population level, change in tuning width was rather small (median percent change: 96.3%). However, this decrease was statistically significant (p = 0.02).

Finally, HWHHs obtained with medium contrast were expected to change little: medium contrast was intended to evoke response close to the mean obtained with the three contrasts in mixed contrasts protocols (although slightly lower by experimental design), therefore inducing comparable amount of adaptation. However, cumulative distributions do not overlap (Fig. 11A, circles) and there appears to be a significant, although small, broadening of the tuning curves after adaptation to the medium contrast (median 105%, n = 61, p = 0.02). At the single cell level, significant increase in tuning width was observed in 16.4% of the cells (10/61).

The conclusion up to this point is that orientation-tuning width appears to be adjusted by contrast adaptation. Adapting to a high contrast stimulus slightly reduced the HWHH compared to a situation in which stimuli had a lower (on average) contrast. An opposite and quite stronger effect occurred with low contrast stimuli: when adapted to a higher (on average) contrast, responses to low contrast stimuli were depressed and tuning widths were thinner. Conversely, adapting to low contrast allowed for recovery from adaptation to a higher (on average) contrast, and this resulted in both an increase in response amplitude and an increase in tuning width, though these effects were not correlated on a cell by cell basis. Thus, the way matched adaptation reduced the effects of contrast on tuning width was mostly by increasing tuning width with low contrast stimuli, and to some extent with medium contrast stimuli.

### Effect of contrast on the untuned response component

Fits made to orientation tuning data included a parameter, *y_0_*, which reflects the untuned component in the response of the cells. This term does not correspond to spontaneous activity that was removed prior to the fit. Since response strength varies greatly among cells, we examined, not the *y_0_* itself, but its amplitude relative to the full response height. This measure has previously been named “relative untuned response amplitude” (RURA) [69]. It is expressed, as a percentage, as:
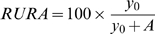



This is complementary to the ‘orientation index’ used in other studies: OI = A/(*y_0_*+*A*). Note that RURA and HWHH represent different facets of orientation selectivity. Depending on contrast, correlation between HWHH and RURA were either weak, or not significant (not illustrated). For high contrast, the correlation (linear) was significant (p<0.0001) but with a low correlation coefficient (r^2^ = 0.135). For medium contrast, the correlation was not significant (p = 0.16). For low contrast, the correlation was significant (p = 0.003) but showed an inverse relationship between the two variables (r = −0.51, r^2^ = 0.26). Cells with broad HWHH may show RURA values close to zero while cells with thin HWHH may show RURA values larger than zero. We also checked for the possibility that the RURA is not cleanly separated from orientation bandwidth, as would occur if orientation tuning is not truly Gaussian, by examining the residuals in a dozen cases. We have not found any systematic structure that would indicate “fatter tails” compared to the Gaussian. Thus, while we cannot exclude that departure from Gaussian may be present in some cases, this does not appear to be a systematic trend.

**Figure 12:**
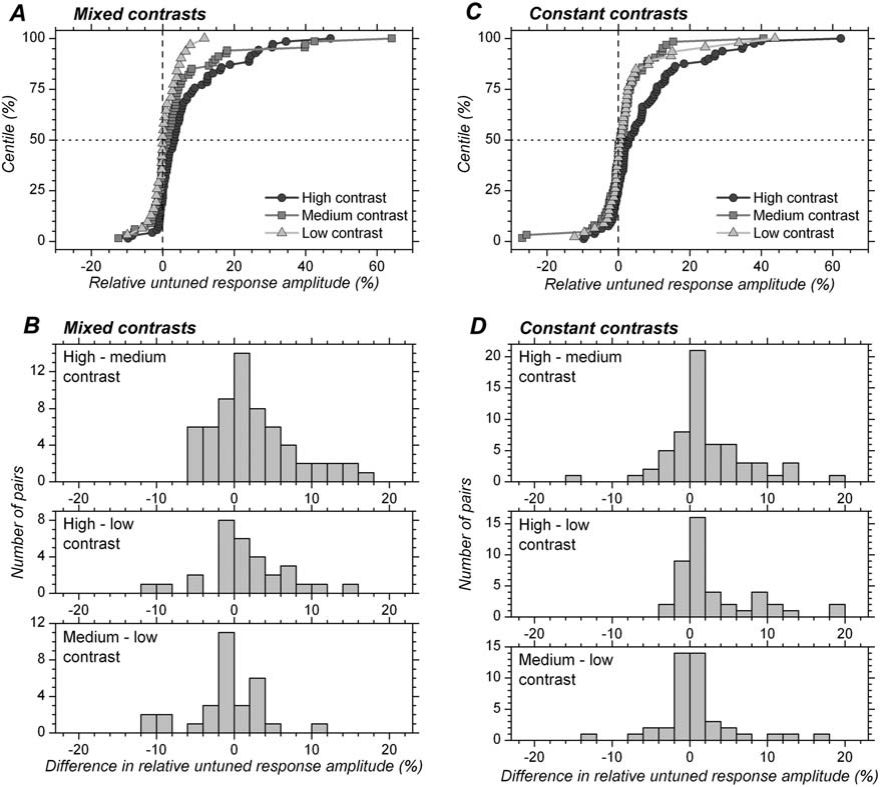
Changes in relative untuned response amplitude with different contrasts. A. Cumulative distribution for the three contrasts in the mixed contrasts condition. The RURA expresses the proportion of response amplitude that lacks orientation selectivity, relative to the total response amplitude. Values close to zero indicate null response to the orientation orthogonal to the preferred one. Values less than zero indicate firing rates lower than spontaneous activity, suggesting cross-orientation suppression. Values larger than zero indicate responses to orthogonal stimuli. B. Distribution of differences in RURA with different contrasts, in the mixed contrasts condition. Upper histogram: RURA obtained with high contrast minus RURA obtained with medium contrast. Middle histogram: RURA obtained with high contrast minus RURA obtained with low contrast. Lower histogram: RURA obtained with medium contrast minus RURA obtained with low contrast. At the population level, a significant difference was observed between high and medium contrast only, with larger RURA, on average, at high contrast. C. Cumulative distributions for each of the three contrasts, for the constant contrast blocks. D. Distribution of differences in RURA with different contrasts, for the constant contrast blocks. RURA values obtained with high contrast were significantly larger than those obtained with either medium (upper histogram) or low contrast (middle histogram). RURA did not differ between medium and low contrasts (lower histogram). We did not test differences in RURA at the single cell level as RURA calculation combines two parameters, each with its own associated standard error.

In most cells, the RURA represented only a small percentage of the total response amplitude (Fig. 12A,C, Table 1). A large number of cells showed values very close to zero. In a small number of cases, values are <0%, indicating firing rate reduction below spontaneous activity level by orientations perpendicular to the optimal. However, in both mixed and constant contrasts conditions, a significant proportion of cells showed a RURA>10% at high and medium contrast (Fig. 12A,C). Cumulative distributions shown in Fig. 12A,C suggest a tendency for RURA to increase with contrast, which was tested in a pairwise fashion. Changes in RURA were calculated as RURA_C2_−RURA_C1_, with C1 and C2 two different contrasts (C1<C2), and the distributions of this difference are shown in Fig. 12B,D.

Contrast did change the RURA, when comparing medium and high contrast (p = 0.0035) in the mixed contrasts condition. The median increase in RURA with high contrast was +1.22% (mean +2.32%). This means that, with an increase in contrast, response to non-optimal orientations did increase proportionately *more than expected* given increase in tuned response amplitude. In this case, changes in RURA corroborated changes in HWHH, implying further decrease in orientation selectivity with increase in contrast. On the other hand, RURA did not change significantly when comparing high and low contrast (p = 0.16, median difference 0.36%) or medium and low contrast (p = 0.38, median difference −0.78%).

When considering constant contrast conditions, RURA did not differ significantly between low and medium contrast (p = 0.38): cumulative distributions superimpose almost completely (Fig. 12C, Table 1) and differences showed a median value (0.06) close to zero (Fig. 12D bottom; Table 2). On the other hand, RURA values obtained with high contrast were significantly larger – implying less selectivity – than those obtained with either medium (p = 0.0008) or low contrasts (p = 0.0006). In particular, RURA>10% was found in 28% of the cells with high contrast, but in only ∼10% of the cells with low or medium contrasts (Fig. 12D). At the population level, high contrast added 1.25% (median) of untuned response to the full response compared to medium contrast, and 1.28% compared to low contrast responses (Fig. 12D, Table 2) (means were, respectively, 2.48% and 3.10%). It therefore appears that, unexpectedly, contrast adaptation did not reduce, but rather increased the differences between contrasts for this variable.

### Comparison of RURA for one given contrast, with and without matched adaptation

In contrast to HWHH, the RURA showed almost no difference between matched and unmatched adaptation. RURA for low and medium contrasts were not significantly modified by adaptation (not illustrated). For high contrast, there was a moderate but significant decrease in RURA (p = 0.046; median difference: −0.79%, mean difference: −1.41%) with adaptation to high contrast compared to adaptation to mixed contrasts (not illustrated). Thus, the relative proportion of unselective response appears to be slightly reduced after adaptation to high contrast.

### Relation to receptive field type

Spread in the distributions of changes in HWHH or RURA with changes in contrast indicates important heterogeneity in the behavior of individual neurons (Fig. 8, 9, 12). We examined whether this heterogeneity could be related to differences in other cell properties. The first property we studied was the RF types of the cells, that have been classified as “simple” or “complex” using the “relative modulation” (see Methods) in 61 of the 87 orientation selective cells analyzed in this study. Sixteen cells were simple (26%) and 45 complex (74%). These proportions appear similar to those previously reported for marmoset V1 [46]. Increases in HWHH with increases in contrast did not differ between simple and complex cells, in mixed as well as in constant contrast blocks (Mann-Whitney U test, p>0.05). Similarly, changes in RURA consequent to changes in contrast did not depend on RF type (p>0.05).

### Relation to contrast range and to contrast sensitivity

We next examined whether heterogeneity between cells with respect to the effect of contrast on orientation tuning could be related to differences in their contrast sensitivities and to the actual contrast values that were used.

CRFs were quantified using the hyperbolic ratio equation in 81 cells (see Methods; not illustrated). The half saturation constant (*C_50_*) was 27.7% (median; interquartile: 17.6%). There was no significant correlation between *C_50_* and changes in HWHH or changes in RURA induced by changes in contrast. The exponent of the CRF (*n*) was 3.1 (median; interquartile: 1.8). There was also no significant relationship between changes in tuning width and the exponent of the CRFs. Changes in orientation tuning with contrast therefore are not related to the contrast sensitivity of the cells.

Changes in HWHH or changes in RURA were also unrelated to the actual range of contrast used: depending on cell sensitivities, the ratios of high/low contrasts we used ranged between 2 and 5.6. However, changes in HWHH were not correlated with these ratios.

### Relation to receptive fields eccentricity

Sixty out of the 114 cells examined in this study were recorded in the operculum (eccentricity <3 deg) and 54 in the calcarine (eccentricity between 6 and 16 deg, most around 7–8 deg). Studies showing contrast-invariance of orientation selectivity were usually based on recordings obtained parafovealy. It was therefore possible that our discrepant result could be due to a different behavior for neurons with RFs located at larger eccentricity.

Whether recordings were obtained in the calcarine or in the operculum did not affect the proportion of orientation selective and non-selective cells (p = 0.27, Chi^2^ test). Orientation selective cells represented 44/54 cells (81.5%) in the calcarine and 43/60 cells (71.7%) in the operculum.

When considering HWHH of orientation tuning curves obtained in the *mixed contrasts blocks*, there did not appear to be any significant difference between opercular and calcarine recordings (Mann-Whitney U test, p = 0.12 for high contrast, p = 0.09 for medium contrast and p>9.99 for low contrast; not illustrated). However, HWHH obtained in *constant contrast block* did show a near significant difference between opercular and calcarine recordings (p = 0.05 for high contrast, p = 0.06 for medium contrast and p = 0.05 for low contrast). Median HWHH for opercular recordings were 25.9 (n = 40), 23.9 (n = 27) and 20.6 (n = 19) deg for high, medium and low contrasts respectively. For calcarine recordings, median values were 19.9 (n = 40), 18.4 (n = 36) and 13.2 (n = 27) deg, respectively. Confirming this trend will require a larger sample.

Nevertheless, decreases in orientation tuning width with decreases in contrast were not related to the recording sites (not illustrated). In mixed contrasts blocks, percent changes in HWHH were not significantly different between the calcarine and the operculum (Mann Whitney U test, medium vs. high contrast: p = 0.70; low vs. high contrast: p>0.99; low vs. medium contrast: p = 0.72). Percent changes in HWHH also did not differ significantly in the case of constant contrast blocks (medium vs. high contrast: p = 0.37; low vs. high contrast: p = 0.07; low vs. medium contrast: p = 0.86). This indicates that the lack of contrast invariance observed with briefly flashed stimuli is not the consequence of some peculiar behavior for neurons with RFs located at relatively large eccentricity.

### Relation to cortical layers

We also examined orientation selectivity and the effects of contrast on orientation tuning with respect to the layers in which recordings were obtained (n = 94 single-units, 74 orientation selective, 20 not orientation selective).

We first compared orientation tuning width and RURA obtained for cells recorded in supragranular layers (n = 22 cells), layer 4B and 4Cα (pooled together, n = 16), and infragranular layers (n = 36) (unfortunately layer 4Cβ could not be included in this comparison; only two single-units could be isolated in this layer, one that was not visually responsive and the other that was not orientation selective). We did not find significant differences, whatever the contrast and stimulation condition (not illustrated). This agrees with quantitative studies that also failed to reveal profound differences in orientation selectivity between layers in macaque V1 [e. g., 70].

We next examined whether the effects of contrast on orientation tuning width and RURA differed between layers. We found that contrast affected tuning width and RURA in a similar fashion when comparing layers 4Cα and 4B with infragranular and supragranular layers (not illustrated). Lack of contrast-invariance of orientation tuning cannot therefore be attributed to a difference between neurons relaying magnocellular inputs (layers 4Cα and 4B) and neurons possibly receiving convergent magnocellular and parvocellular inputs (supragranular and infragranular layers).

## Discussion

The two main results of this study are: 1) Orientation tuning does not appear to be contrast-invariant when stimuli vary in both contrast and orientation at a high rate. 2) Orientation tuning is less affected by contrast when neurons are given enough time to adapt to one particular contrast, suggesting that contrast adaptation plays a role in contrast-invariance of orientation tuning.

### Contrast adaptation may contribute to contrast-invariance of orientation tuning

Contrast-invariance of orientation tuning has been demonstrated in a large number of studies in cats [6]–[9], [11], [13], ferrets [10] and squirrels [12]. In almost all these studies, stimuli consisted of drifting sine-wave gratings that were presented for relatively long durations −4 seconds usually. This long stimulation duration makes it possible that contrast adaptation was recruited and could have contributed to contrast-invariance of orientation tuning. One exception is the study by Li and Creutzfeldt (1984) [7] in which drifting light bars were used as a stimulus. However, in this study, contrasts were not randomized; one single contrast was used for one block of bar presentation, such that adaptation to that contrast likely occurred.

Contrast adaptation manifests itself as a slow adjustment of neural firing rate during the prolonged presentation of a stimulus of constant contrast. During prolonged presentation of a high contrast stimulus, firing rate progressively decreases with a time course of seconds [29], [31]–[36], [71]–[73]. With drifting stimuli, many cells in V1 display adaptation time constants that are less than 4 seconds [31]–[37]. With flashing stimuli, adaptation appears to be faster than with drifting stimuli [65], [66; and present study]. Conversely, after cessation of the high contrast stimulus, response to low contrast stimuli is initially depressed, and progressively recovers with a time course of seconds to tens of seconds [29], [31], [33]–[35], [74].

Contrast adaptation could therefore play a significant role during the 4-second stimulus presentation that was typically used in studies of contrast invariance. This has led us to examine whether contrast-invariance of orientation tuning still holds with briefly flashed stimuli. We thus had two purposes in mind: determining whether contrast-invariance of orientation tuning still occurs when the stimulus presentation time is commensurate with the fixation duration observed in natural viewing conditions (200–300 msec) [41]–[45]. And, if not, determining whether contrast adaptation, a relatively slow phenomenon compared to fixation duration, contributes to contrast-invariance of orientation tuning.

Our results show that orientation tuning is not contrast-invariant with briefly flashed stimuli, when the contrasts do not match the contrast to which the cells are adapted. We also found that contrast-invariance of orientation tuning was partially restored when stimuli had a contrast corresponding to the contrast to which the cells have been adapted. However, this restoration was not complete and orientation-tuning curves obtained with the lowest contrast still appeared to be slightly thinner, on average, than those obtained with the highest contrast.

### How contrast adaptation might contribute to contrast-invariance of orientation tuning

Mechanisms responsible for contrast adaptation have been examined in several studies. Thalamic neurons do not present strong contrast adaptation when stimulated with sine-wave gratings drifting with a low temporal frequency (<3 cy/sec) [29], [34], [35], [74]–[78]. In parallel, in cortical neurons with simple RFs, the F1 component of the membrane potential response – the modulation at the stimulus rate, which is most likely derived from the thalamic input – is also only weakly affected by contrast adaptation [35], [79], [80]. This, together with other arguments [e. g., 29], [81]–[83], indicates that contrast adaptation is essentially generated intracortically.

In intracellularly recorded cortical neurons, it is observed that, in contrast to the F1 component, the F0 component (mean membrane potential) shows significant changes with adaptation: high contrast stimulation in cortical neurons is associated with a progressive membrane potential hyperpolarization [35], [73], [80], [84] while cessation of high contrast stimulation is associated with a progressive recovery from hyperpolarization with a time constant of several seconds [35], [73]. This long-lasting hyperpolarization has been shown to depend, at least partially, on intrinsic membrane properties, as high-intensity current injection in single neurons *in vivo* results in a membrane potential hyperpolarization that mimics the one obtained with high contrast visual stimuli, and which is able to reduce responses to visual stimuli [35], [73]. *In vitro* studies showed that this long-lasting hyperpolarization depends largely on the activation of a sodium-dependent potassium current [85], [86]. Channels responsible for this current have recently been cloned and their distributions characterized [87].

Nevertheless, an intrinsic adaptation mechanism cannot explain the fact that adaptation shows some stimulus specificity [31], [66], [72], [74], [75], [88]–[90]. It is also to be noted that changes in visually evoked F0 amplitude can to some extent be ambiguous: they do not distinguish between an intrinsic mechanism and changes in synaptic responses that are not modulated at the temporal frequency of the stimulus, in particular in complex cells. To identify more precisely the relative contribution of intrinsic and synaptic (or network) mechanisms in contrast adaptation, a study recently examined the aftereffects of high contrast adaptation on RFs mapped using a sparse-noise stimulation technique [73]. The results demonstrated the occurrence, sometimes in the same neurons, of both reduced synaptic RF amplitude, and of global membrane potential hyperpolarization. This indicates that both intrinsic and synaptic (or network) mechanisms contribute to contrast adaptation. That both mechanisms contribute is consistent with stimulus dependent adaptation, provided cortical connections are made between neurons displaying different stimulus preferences (for orientation see [91]).

In the temporal domain, the stimuli we used in the present study correspond to square pulses, whose Fourier spectrum necessarily includes high frequencies. Recent studies showed that contrast adaptation could actually occur for fast temporal frequencies in thalamic magnocellular neurons [78]. Therefore, it cannot be excluded that subcortical mechanisms also contributed to the adaptation we observed.

We may now examine how the mechanisms involved in contrast adaptation may influence orientation tuning and its contrast-invariance. Experimental and theoretical studies have identified several possible mechanisms that could contribute to contrast-invariance of orientation tuning. Among these, 3 at least could be modified by contrast adaptation.

The first of these possible mechanisms is inhibition [14]–[17], [19], [21]–[24]. We do not intend to review how different forms of inhibition have been included in different models; suffice it to say that contrast-invariance of orientation tuning requires that inhibition and excitation grow somehow proportionately with contrast. However, although inhibitory neurons possess an intrinsic capacity to adapt similar to that of excitatory neurons [92], the relative amount of excitation and inhibition a cell receives may not be the same at the beginning and at the end of a prolonged stimulus presentation. For example, slow synaptic depression is stronger at excitatory synapses compared to inhibitory synapses *in vitro* [93], [94]. If the temporal dynamics of synaptic responses are not identical for inhibition and excitation, then it is conceivable that the relative amount of excitation and inhibition required to achieve contrast-invariant orientation tuning is reached only after some time of visual stimulation.

Inhibition may be important in setting up contrast-invariance of orientation tuning at the membrane potential level. However, it might not be sufficient to explain contrast-invariant orientation tuning for the spiking response [13], [14], [19]. This may require two other mechanisms on which adaptation could act. They correspond to membrane potential fluctuations (or “synaptic noise”) and to the shape of the input-output relationship of cortical neurons. These two mechanisms are strongly intertwined. Indeed, *in vitro*, in the absence of synaptic noise, the input-output relation of cortical neurons is well approximated by a linear relationship (R = β.V) (e. g., [95], [96]). In a feedforward mechanism of orientation selectivity, this linearity would result in a tuning which is not contrast-invariant (e. g., [9], [13]). However, the presence of membrane potential fluctuations *in vivo*, either spontaneous or stimulus-induced, has the effect of expanding the relationship between voltage and firing rate toward lower voltage values: small membrane potential depolarizations induced by low contrast stimuli and/or by stimuli of less than optimal orientation, which remain subthreshold in the absence of membrane potential fluctuations, could generate significant firing if occurring simultaneously with depolarizing synaptic noise. The input-output relation then takes the approximate form of a power law relationship (R = β.V^α^) [9], [13], [18], [97], [98]. Interestingly, Finn et al. (2007) [13] found that noise amplitude tends to increase when contrast decreases, resulting in an adjustment of the power law for different contrasts, allowing the preservation of contrast-invariance of orientation selectivity. In their theoretical study, Hansel and van Vreeswijk (2002) [18] found that contrast-invariance of orientation tuning may be obtained for some restricted ranges of noise level. Contrast-invariance would be lost for noise levels that are either above or below this optimal range. The fact that adaptation partially restores contrast-invariance of orientation tuning, as shown in the present study, might therefore be explained if the amplitude of membrane potential fluctuations is also dynamically adjusted by adaptation. This is certainly possible, as membrane potential fluctuations likely result from network interactions (e.g., [99], [100]) involving neuronal elements that exhibit contrast adaptation.

The third mechanism involved in contrast-invariance of orientation selectivity, which may be modified by adaptation, is the input-output power law itself. Even if the amplitude of membrane potential fluctuations remains constant, it is expected that the slow membrane potential changes induced by adaptation will result in the modification of one or the other power law parameters (slope or exponent). This dynamic adjustment might also be involved in the generation of contrast-invariant tuning. The effects of contrast adaptation on membrane potential noise and on input-output relationship in single cortical neurons are currently under examination.

### Species differences

Although contrast adaptation did reduce contrast-dependent differences in orientation tuning, it did not completely suppress them. There was still a significant difference in tuning width between the lowest and highest contrast, such that, despite adaptation, orientation tuning was still not completely contrast-invariant. It is possible that this reflects a difference between flashing and drifting stimuli, involving distinct networks, or different interaction dynamics within these networks.

Alternatively, it could represent a species difference (note however that a recent study [13] reported occurrences of tuning width reduction with low contrasts in the cat). It is also worth noting that, in the cat, not only orientation tuning, but also spatial frequency tuning is largely independent of stimulus contrast [5], [8]. On the other hand, spatial frequency tuning *does* vary with contrast in the primate [101]. We first hypothesized that species difference in contrast-invariance of orientation tuning finds its roots in two fundamental differences between cat and monkey visual processing streams. In cat, X- and Y-cells show relatively small differences in contrast sensitivity [3], [102]–[106] and both afferent streams target cells that are orientation selective [107]. On the other hand, in the primate, neurons in the magnocellular pathway show much higher contrast sensitivity than those in the parvocellular pathway [108]–[111]; in addition, parvo- and magnocellular inputs target cells with different orientation selectivity: neurons in the parvocellular recipient layer 4Cβ show weak or no orientation selectivity whereas neurons in the magnocellular recipient layer 4Cα show sharp orientation tuning [112]–[114]. It is therefore conceivable that, at low contrast, mostly orientation selective cells of layer 4Cα were activated, whereas at high contrast both orientation and non-orientation selective neurons are activated in both layer 4Cα and layer 4Cβ. Beyond layer 4C, a large number of cells receives converging inputs from both parvo- and magnocellular pathways [115], [116]. Thus, by changing the proportion of orientation selective and non-orientation selective neurons, contrast might have changed orientation-tuning width in these second order cells, which would have been more orientation selective at low than at high contrast. This hypothesis could also provide an explanation as to why RURA increased with contrast. Unfortunately, we did not find a significant difference in contrast-invariance between layers receiving presumably pure magnocellular inputs (layers 4Cα and 4B) and layers where a proportion of neurons may receive converging magnocellular and parvocellular inputs (supra- and infragranular layers). It is possible, however, that magno- and parvocellular inputs are already mixed in layer 4B [117]. An alternative way to examine the implication of parvo- and magnocellular inputs on the effects of contrast on orientation tuning would be to examine orientation-tuning dynamics. In area V1, parvocellular inputs appear to be delayed relative to magnocellular inputs by approximately 20 msec [118]. Given this latency difference, we predict that neurons receiving convergent magno- and parvocellular inputs should show a short latency response appearing with low contrast and displaying sharp orientation selectivity, followed by a more sustained response that should require higher contrast and should show less orientation selectivity.

Another possible explanation for a cat vs. primate species difference is related to their differing CRFs. One of the mechanisms that may contribute to contrast-invariance of orientation tuning, reported by Finn et al. (2007) [13] in the cat, is a compression of the CRF at the cortical level, in comparison to that in the LGN. Their median *C_50_* for the F1 potential in simple cells was only 7.6%. Since *C_50_* for spiking and membrane potential responses are comparable [119], this value may be compared to the median we obtained with spiking responses in the marmoset, which was 27.7%. This indicates that the CRF in the marmoset shows considerably less saturation than in the cat. According to the model of Finn et al. (2007) [13], this lower amount of saturation should result in less contrast-invariance of orientation tuning.

Whatever the reason for the presence of an effect of contrast on orientation tuning, our results are not simply explained by an iceberg effect. Changes in orientation tuning with contrast, in both constant and mixed contrasts conditions, were never proportional to changes in response strength (Fig. 10). This lack of positive correlation suggests that there might be several gain-control mechanisms at multiple stages of the visual pathway, some influencing response amplitude only, and others also influencing mechanisms involved in orientation tuning.

### Effect of contrast on orientation tuning and orientation discrimination

Contrast-invariant orientation tuning, as initially reported in the cat, provided a neuronal correlate to the observation that, behaviorally, orientation discrimination appeared to be contrast-invariant [8], [120], [121]. However, not all psychophysical studies agree on this point; other studies showed that reducing contrast impairs orientation discrimination [122]–[127]. This apparent contradiction has been explained by studies [128], [129] showing that interaction between contrast and orientation discrimination actually depends on stimulus size: orientation discrimination thresholds were found to be elevated at low contrast for small stimuli, but were found to be independent from contrast provided stimuli were large enough.

Models suggest that orientation discrimination depends on the differential activity of orientation detectors [66], [68], [120]–[122], [130]–[133]. Thus, orientation discrimination should improve when orientation bandwidth decreases. Behaviorally observed increases in discrimination threshold at low contrast is therefore opposite to what would be expected given our finding that neurons are more selective (decreased tuning width) at low contrast. However, tuning width is not the sole relevant variable in orientation discrimination. Noise also probably plays an important role [66], [68], [120]–[122], [130], [131], [133]. In our results, relative decrease in tuning width with contrast was less than relative decrease in response strength. Given that noise grows proportionately with response strength, this implies that the reduction in tuning width at low contrast may not be sufficient for maintaining orientation discrimination thresholds comparable to those obtained at higher contrasts. It nevertheless remains possible that orientation discrimination at low contrast would be even worse if orientation tuning was contrast-invariant.

### Consequences of contrast adaptation

At the behavioral level, contrast adaptation leads to a variety of effects: thresholds are elevated for detecting stimuli similar to the adapting stimulus [81], [82], [122], [132], [134]; the apparent contrast of suprathreshold stimuli appears to be decreased [135]–[137]; and test stimuli appear to be repelled from the adapting stimulus as in motion (see [138] for an historical account), tilt [139] or size [140] aftereffects. Although these different effects are consistent with the presence of psychophysical “channels” commensurate with the selectivity of individual cortical neurons, they do not reveal the function of contrast adaptation.

The functional role of contrast adaptation has remained somehow elusive. At one extreme, it may be proposed that contrast adaptation does not have much of a functional role in vision; contrast adaptation may simply be the by-product of the presence of sodium-dependent potassium channels, whose main functions may be to provide a “metabolic gate-keeper” and to protect cells against ischemic stress [141], [142]. At the other extreme, it has been proposed that adaptation (to contrast as well as to other stimulus dimensions) has a fundamental functional role, which is to maximize information transmission [143]–[147].

Neurophysiological studies have suggested that contrast adaptation results in an adjustment of the (limited) dynamic range of the neurons around the adapting contrast [31], [34], [36], [74], [75], [89], [148]. This should be manifested behaviorally by an improvement in contrast *discrimination* around the adapting contrast. However, behavioral testing failed to show such a beneficial effect [149], [150] or showed it to be limited to extreme values of adapting and test stimuli contrast [151], [152].

Benefits of contrast adaptation have therefore been sought with respect to other stimulus dimensions. With respect to spatial frequency for example, a psychophysical study [153] showed that adaptation with natural images results in a “whitening of the neuronal image”, that is, in a balancing of our sensitivity to spatial frequencies, despite the dominance of low spatial frequencies in natural scenes. With respect to temporal frequency, studies suggest that contrast adaptation might reduce signal redundancy in the temporal domain [154].

Finally, psychophysical studies have shown that adapting to a high contrast stimulus of *constant* orientation does improve orientation discrimination when test and adapting orientation differ by 10–20 deg [122], [132]. This result has been extended to the single neuron level in neurophysiological studies [66]. One possible explanation for this improvement is the repulsive shifts in preferred orientation of the cells relative to the orientation of the adapting stimulus [66], [90], [155] – But see [156]). Improvement might also involve changes in signal-to-noise ratio [66]. However, in the studies mentioned above, the adapting stimulus was presented at one orientation only. Our results suggest one additional possibility, which is that high contrast adaptation also results in sharpening orientation tuning, relative to mismatched adaptation. It would therefore be of interest to examine whether adaptation, using adapting stimuli with quickly changing orientation, as in our protocol, or using natural scenes that contain a wide range of orientations, also improves orientation discrimination.

### Conclusion - Contrast invariance in general

Our results suggest that, when stimuli are presented for a duration comparable to that of a visual fixation, and without prior adaptation to the contrast of these stimuli, contrast-invariance of orientation selectivity breaks down. This indicates that contrast-invariance of orientation tuning is not instantaneous and rather requires some time of adaptation to the prevailing contrast to be expressed. In fact, there appear to be a number of response properties that do not show contrast-invariance. One is spatial frequency tuning in primate [101] – but not in cat. Another is direction selectivity, which improves when contrast decreases [157]. A third one is the optimal stimulus size, which increases when contrast decreases [58]–[60]. Yet, studies have shown that we are well able to perceive and identify objects after a processing duration that is even shorter than a fixation duration (e.g., [158], [159]), even when image contrast is severely reduced [160]. Altogether, these results suggest that accurate and stable detection, categorization and recognition of visual objects is possible in the face of ever changing stimulus intensity, even in the absence of contrast-invariance for V1 neurons selectivity.
